# Livestock production losses attributable to brucellosis in northern and central Tanzania: Application of an epidemiological-economic modelling framework

**DOI:** 10.1371/journal.pntd.0012814

**Published:** 2025-02-14

**Authors:** Ângelo J. F. Mendes, Daniel T. Haydon, William A. de Glanville, Rebecca F. Bodenham, AbdulHamid S. Lukambagire, Paul C. D. Johnson, Gabriel M. Shirima, Sarah Cleaveland, Emma McIntosh, Nick Hanley, Jo E. B. Halliday

**Affiliations:** 1 School of Biodiversity, One Health and Veterinary Medicine, College of Medical, Veterinary and Life Sciences, University of Glasgow, Glasgow, United Kingdom; 2 Kilimanjaro Clinical Research Institute, Kilimanjaro Christian Medical University College, Moshi, Tanzania; 3 School of Life Sciences and Bioengineering, The Nelson Mandela African Institution of Science and Technology, Arusha, Tanzania; 4 School of Health and Wellbeing, College of Medical, Veterinary and Life Sciences, University of Glasgow, Glasgow, United Kingdom; Makerere University, UGANDA

## Abstract

Livestock brucellosis is an endemic disease in many low-resource settings. Despite its widespread distribution, little is known about the scale of economic impacts caused by the disease. This study aimed to develop an integrated epidemiological-economic modelling framework to estimate production losses attributable to livestock brucellosis, using Tanzania as a case study. Data on livestock production and prevalence of exposure to *Brucella* spp. were obtained from surveys conducted in northern and central Tanzania between 2013 and 2019. A clustering algorithm was applied to classify households into pastoral and non-pastoral production systems. A Bayesian latent-class analysis model was applied to derive livestock brucellosis prevalence estimates. A herd-growth model was used to estimate production losses attributable to brucellosis. A total of 1,541 households (384 classified as pastoral and 1,157 as non-pastoral) contributed data on livestock production or prevalence of exposure to *Brucella* spp. The median (95% uncertainty interval, UI) individual-level brucellosis prevalence in cattle, sheep, and goats was 5.1% (3.4–6.9), 1.3% (0.1–3.0), and 2.5% (0.3–4.8) in the pastoral system, and 0.7% (0.1–1.6), 1.6% (0.2–3.8), and 2.5% (0.3–4.9) in the non-pastoral system, respectively. The median (95% UI) annual losses attributable to brucellosis in cattle, sheep, and goats, per infected animal, were 74.4 (26.2–211.7), 9.7 (3.4–23.1) and 10.6 (3.7–25.0) international dollars (int. $) in the pastoral system, and 62.3 (16.8–228.6), 6.3 (1.8–17.1) and 7.0 (2.2–17.9) int. $ in the non-pastoral system, respectively. Household-level losses were equivalent to 4.4% (2.1–8.8) and 0.6% (0.2–1.6) of the median (95% UI) livestock-derived income in the pastoral and non-pastoral systems, respectively. This study did not capture the system-wide impacts of brucellosis, including on human health. The estimated losses are only a part of the full societal economic impact of the disease. These results can be used to inform cost-benefit analyses of potential interventions and guide policy development for brucellosis control.

## Author summary

Brucellosis is a disease caused by bacteria of the genus *Brucella*, which can infect humans and animals. The disease is globally distributed, but most infections occur in livestock-dependent communities of low-resource settings. In this study, we developed and applied a new mathematical model to estimate production losses attributable to livestock brucellosis in northern and central regions of Tanzania. We obtained data on brucellosis prevalence and livestock production characteristics from several sources, including household surveys. We then analysed these data using a modelling approach that can estimate the difference in the value of livestock production (e.g., animals sold or slaughtered, milk produced) between the current situation of brucellosis presence and a hypothetical scenario of brucellosis absence. This difference in the value of livestock production was defined as production loss attributable to brucellosis. We found that brucellosis causes considerable production losses in the study area. In households that keep livestock as their primary means of subsistence, annual losses amounted to 4.4% of the income derived from livestock. However, there was significant heterogeneity in estimated losses across production systems and geographical areas. These results can help inform further research and guide the development of brucellosis control policies.

## Introduction

Livestock play a key societal role in low-resource settings, providing nutrition, fertiliser, and transportation, as well as providing a means of accumulating financial and social capital [1,2]. However, livestock diseases compromise these functions, leading to socioeconomic deprivation, food insecurity, and poor human health and well-being [3].

Livestock brucellosis is endemic in many low- and middle-income countries, where it causes relatively low mortality, but high reproductive morbidity (e.g., abortion, infertility, and sterility) in animals [4]. Many of the symptoms of livestock brucellosis are non-specific, transient, or insidious, which may lead to poor recognition of disease impacts and low motivation for disease control by livestock keepers, veterinary authorities, and policymakers [5–7].

Estimating production losses attributable to brucellosis in endemic areas is important to help inform and justify investment in disease control interventions, which take into account the perspectives of both the payers and beneficiaries. However, this task is challenging for several reasons. First, there are limited available data on livestock brucellosis prevalence, geographical distribution, and direct physiological impacts, such as the probabilities of abortion or perinatal mortality in infected animals [8–11].

Second, in each country or region, the prevalence and societal impacts of livestock brucellosis vary depending on the production system. For instance, brucellosis is endemic in Tanzania, a country with one of the largest livestock populations in the African continent [12–14]. In northern regions of the country, the prevalence of bovine brucellosis is much higher in the pastoral system (6.3%; 95% Bayesian credibility interval, BCI: 4.5–9.0) than in the non-pastoral system (0.9%; 95% BCI: 0.1–2.1) [15]. Studies aiming to estimate production losses attributable to livestock brucellosis must consider the differences in prevalence and management practices between these systems [16]. Evidence of the relative losses attributable to brucellosis in pastoral and non-pastoral production systems may help support the planning, design, and prioritisation of potential control interventions.

Finally, there is a lack of conceptual and methodological standardisation in the livestock disease impact estimation literature. The terms ‘loss’ and ‘cost’ are often used interchangeably in the context of livestock brucellosis impacts [17,18]. In this study, ‘loss’ refers to production potential not realised because of the disease, while ‘cost’ encompasses both ‘loss’ and disease-related expenditure [19,20]. Additionally, the methods applied to estimate the impacts of livestock disease are highly heterogeneous. This heterogeneity is especially problematic in the case of brucellosis because many methods do not capture the potentially considerable foregone losses to livestock keepers that result from reproduction failure (e.g., the production potential of an unborn calf) [21]. This lack of methodological standardisation is a key problem being addressed by the Global Burden of Animal Diseases (GBADs) programme [22].

Despite the clear challenges in defining and measuring livestock brucellosis impacts, the currently available evidence suggests that livestock brucellosis is likely to cause significant income loss in livestock-keeping households of low-resource settings [23]. In Brazil, India, Sudan, Kenya, and Uganda, the estimated annual cost of bovine brucellosis per infected animal ranged between 126 and 734 international dollars (int. $) (currencies converted using World Bank conversion rates available at https://data.worldbank.org) [17,18,24–28]. Comparatively lower estimates have been reported in small ruminants. In India and Iraq, the estimated annual cost of brucellosis in sheep or goats per infected animal ranged between 27 and 111 int. $ (currencies converted as indicated above for bovine brucellosis) [24,29,30].

This study aimed to develop an integrated epidemiological-economic modelling framework to estimate the production losses attributable to livestock brucellosis in endemic settings. This framework was applied in the context of northern and central Tanzania to estimate the losses attributable to livestock brucellosis in pastoral and non-pastoral systems separately. Three levels of production losses were considered: i) at the individual level, i.e., the losses per infected animal; ii) at the household level, i.e., the losses per livestock-keeping household; and, iii) at the regional level, i.e., the sum of household losses in each region. Household expenditure due to livestock brucellosis (e.g., expenditure on treatment) is likely minimal in the study area and was not considered.

## Materials and methods

### Ethics statement

This study used data from the ‘Social, Environmental, and Economic Drivers of Zoonotic disease’ (SEEDZ) project. Approval for the SEEDZ study was provided by the ethics review committees of the Kilimanjaro Christian Medical Centre (KCMC/832) and National Institute of Medical Research (NIMR/2028) in Tanzania, and by the ethics review committee of the College of Medical, Veterinary and Life Sciences at the University of Glasgow (39a/15) in the UK. Formal written consent was obtained from all SEEDZ participants.

### Framework overview and study area

A framework was developed to: i) optimise the use of currently available data, capturing uncertainty in livestock production and brucellosis prevalence estimates; ii) account for heterogeneity in livestock production systems across the study area; and, iii) estimate production losses attributable to brucellosis in each system.

The framework was divided into three parts: i) Part I – retrospective data gathering and harmonisation (the process of systematically improving comparability of data collected by multiple studies) [31]; ii) Part II – harmonised household classification models; and, iii) Part III – estimation of production losses due to livestock brucellosis by production system.

This study focused on some of the northern and central regions of Tanzania: Arusha, Manyara, Kilimanjaro, Tanga, Dodoma, Singida, Simiyu, and Mara Regions. These regions cover an area equivalent to approximately 29% of mainland Tanzania but include around half of the livestock population in the country [32,33]. In October 2017, there were 13.0, 10.6, and 3.2 million cattle, goats, and sheep in these regions, respectively, which correspond to 42.6%, 55.8%, and 57.9% of the total population of each species in mainland Tanzania [33].

This large study area has highly diverse socio-economic and geo-climatic settings. The regional average human population density is close to the national average (51 people/km^2^), except in Kilimanjaro, where there are approximately 124 people/km^2^ [34]. Kilimanjaro is also the region with the smallest average household size (4.3 people per household) in the study area, contrasting with Simiyu, where the average household size is the largest nationally (6.9 people per household) [34]. The area is dominated by savannas, grassland and, to a lower extent, small-scale crop cultivation [35]. The mean annual temperature ranges from 20.2 ^o^C in Arusha to 23.2 ^o^C in Tanga [36]. The mean annual precipitation ranges from 616 mm in Dodoma to 1,060 in Mara [36]. The elevation ranges from the sea-level values in the Tanga region to the highest point of the African continent: Mount Kilimanjaro (5,895 m) [37]. Arusha and Manyara are dominated by livestock-only, grassland-based systems, whilst the other regions include predominantly rainfed mixed crop/livestock systems [38].

### Part I – Retrospective data gathering and harmonisation

A protocol based on the Maelstrom Research guidelines for retrospective data harmonisation was followed [31]. An informal review of the literature on household surveys in northern and central Tanzania and classification of livestock production systems was carried out. Researchers with experience in this context and research area were also contacted for feedback on methodological issues. These preliminary steps enabled the identification of candidate surveys and the definition of survey selection criteria.

The SEEDZ project delivered a large-scale, cross-sectional survey, dedicated to the analysis of risk factors for several zoonoses in people and animals, between February and November 2016, in two regions of northern Tanzania: Arusha and Manyara Regions. In the SEEDZ project, households were classified by production system, and livestock were serologically tested for exposure to *Brucella* spp. No previous administration of vaccines against *Brucella* spp. in livestock was reported by participating households. The study design and the methods used have been described in detail elsewhere [15,16]. In the SEEDZ project, a household is defined as a separate dwelling within a compound with a husband, his wife(s) and their children. The SEEDZ survey was used as a contextual, spatial, and temporal reference for gathering data on livestock production from additional surveys.

The following criteria were used for inclusion of additional surveys: (i) surveys that were delivered in the SEEDZ study area (Arusha and Manyara) or neighbouring regions (Kilimanjaro, Tanga, Dodoma, Singida, Simiyu, and Mara), (ii) surveys that collected information on the number of livestock kept and their production characteristics (e.g., wholesale price, milk yield, lactation length, and milk price), household demographics, crop agriculture, livestock management practices, food consumption practices, and indicators of household vulnerability (e.g., crop losses, illness in livestock and people), (iii) surveys that took place between 2013 and 2019 (three years before and after SEEDZ), and (iv) had publicly accessible survey data that included approximate geolocation of the households.

The application of survey selection criteria led to the inclusion of the following surveys, in addition to SEEDZ: i) the ‘Living Standards Measurement Study’ (LSMS), and ii) the ‘Rural Household Multiple Indicator Survey’ (RHoMIS). General information about these surveys, including research design, data collection procedures, questionnaires, and data processing and evaluation methods, was documented. These surveys gathered data through face-to-face interviews with either household heads or other household members.

The LSMS is a multi-country, nationally-representative household survey programme led by the World Bank since the 1980s [39]. This programme has a longitudinal design, which allows researchers to monitor the living conditions of a sample of households over time in ‘waves’. In Tanzania, four waves of the programme have been delivered until 2019 [40]. Data from waves three (LSMS3, October 2012 to November 2013) and four (LSMS4, October 2014 to November 2015) were used in this study due to their temporal proximity to the SEEDZ survey. In the LSMS project, a ‘household’ is defined as ‘people who live together and share income and also basic needs’ [41,42].

RHoMIS is a standard survey tool focused on household socioeconomic characteristics, which has been applied in more than 30 countries since 2015 [43]. In northern and central Tanzania, based on GPS location data, two applications of the RHoMIS tool were available by June 2020: i) RHoMIS1 - a survey of households randomly selected from community animal health service records in 2017; and, ii) RHoMIS2 - a survey of households randomly selected from village lists obtained from village elders in 2018. In the RHoMIS project, a ‘household’ is defined as people ‘who usually live, sleep and eat’ at a house or compound and includes ‘anyone who is temporarily away for less than 3 months, or anyone who is staying for more than 3 months’ [43].

The definition and availability of variables across survey datasets were compared and analysed to enable an initial evaluation of the potential for running harmonised household classification models, i.e., clustering models based on de Glanville et al. (2020) [16] applied to households from candidate surveys and those in SEEDZ. The clustering analyses of the SEEDZ dataset by de Glanville et al. (2020) [16] served as a basis for data harmonisation and identification of livestock production systems in candidate datasets. The timing, spatial extent, and number of households included in each dataset are available in Table A in Supporting information (S1 File). The spatial distribution of households in each dataset is shown in Fig A in Supporting information (S1 File).

For all datasets, the common variables used for clustering were divided into eight domains (as per de Glanville et al., 2020) [16]: 1) local household environment; 2) household demographics; 3) crop agriculture; 4) numbers of cattle, sheep, and goats owned; 5) other livestock owned; 6) livestock management practices; 7) household food consumption practices; and, 8) indicators of household vulnerability. Domains 1 (local environment) and 4 (number of livestock owned) include only numerical variables. The other domains include only categorical variables (binary variables that indicate the presence or absence of an attribute).

Data on variables in domain 1 (local household environment) were sourced from third-party datasets. A summary description of each environmental variable (and data sources) is available in Table B in Supporting information (S1 File). The methods used to gather these variables were adapted from de Glanville et al. (2020) [16] for SEEDZ. However, some methods were not applicable due to the relatively low precision of GPS locations of households in the candidate surveys. For such cases, buffers were created around the approximated GPS locations of households using the ‘sf’ package [44,45] in R [46]. The buffer radii were set at 5 km for LSMS households in rural areas, 2 km for LSMS households in urban areas, and 1.1 km for all RHoMIS households. These radii were chosen based on the coordinate modification strategy in each survey, which was applied to preserve confidentiality. The mean data value for environmental variables within each buffer was extracted using the ‘exactextractr’ package [47] in R. The proportion of the buffer area classified as each landcover type was extracted using the ‘sf’ package in R. The village area to which the households belonged was calculated as the weighted mean of the areas of the villages covered by the buffer.

### Part II – Harmonised household classification models

de Glanville et al. (2020) [16] applied multiple factor and hierarchical clustering analyses to classify SEEDZ households as pastoral, agro-pastoral, or smallholder. Multiple factor analysis (MFA) enabled the detection of underlying patterns among variables organised in domains [48]. Hierarchical clustering analysis used the outputs of MFA to group SEEDZ households into production systems based on similarities between households. The goal in the current study was to apply the same methodology to classify LSMS and RHoMIS households. However, many of the variables in SEEDZ were not available in LSMS and RHoMIS. The SEEDZ dataset included in de Glanville et al. (2020) [16] had 76 variables. Out of these 76 variables, 12 (15.8%) were absent in LSMS, and 28 (36.8%) were absent in RHoMIS. All the variables that were absent in LSMS were also absent in RHoMIS.

Given differences in availability of variables between datasets, several household classification models were run with different combinations of datasets and variables. Three main integration options were tested: i) adding one non-SEEDZ household at a time to the original model (M0), which initially contained SEEDZ data only, as [16]; ii) adding one non-SEEDZ dataset at a time to M0; or, iii) adding all data together.

The performance of each model was evaluated with reference to the classification of SEEDZ households derived with M0. Given that the purpose of this study was the classification of households into two predefined production systems (pastoral and non-pastoral), agro-pastoral (cluster two) and smallholder (cluster three) households were merged and characterised together as non-pastoral households. The performance metric used was the probability of a model classifying a SEEDZ household correctly as pastoral or non-pastoral. The best-performing model was selected for classifying LSMS and RHoMIS households by production system. The household classification algorithm is described in detail in Supporting information (S2 File).

### Part III – Estimation of production losses due to livestock brucellosis by production system

Part III of this study had two main objectives: i) to gather system-specific demographic, brucellosis prevalence, and production parameter values from the three survey datasets; and, ii) to run herd production simulations in the presence and in the absence of brucellosis, using literature-based information on disease impacts and additional production characteristics. The losses attributable to brucellosis were calculated as the difference between the estimated value of livestock production (including all revenue-generating elements captured in market value and other benefits, such as own consumption of animal products) simulated in the absence and in the presence of the disease. The same workflow, i.e. a series of steps and processes for data analysis, was used to estimate losses in cattle, sheep, and goats. However, whilst only offtake (wholesale, home slaughter, or gifts given away) and milk contributed to production or income from sheep and goats, draught power and dung (e.g., to be used as manure, cooking fuel, and in construction) were also considered in cattle. Indirect consequences of production losses on value-added products such as yoghurt or cheese were not captured in this study.

**Fig 1:**
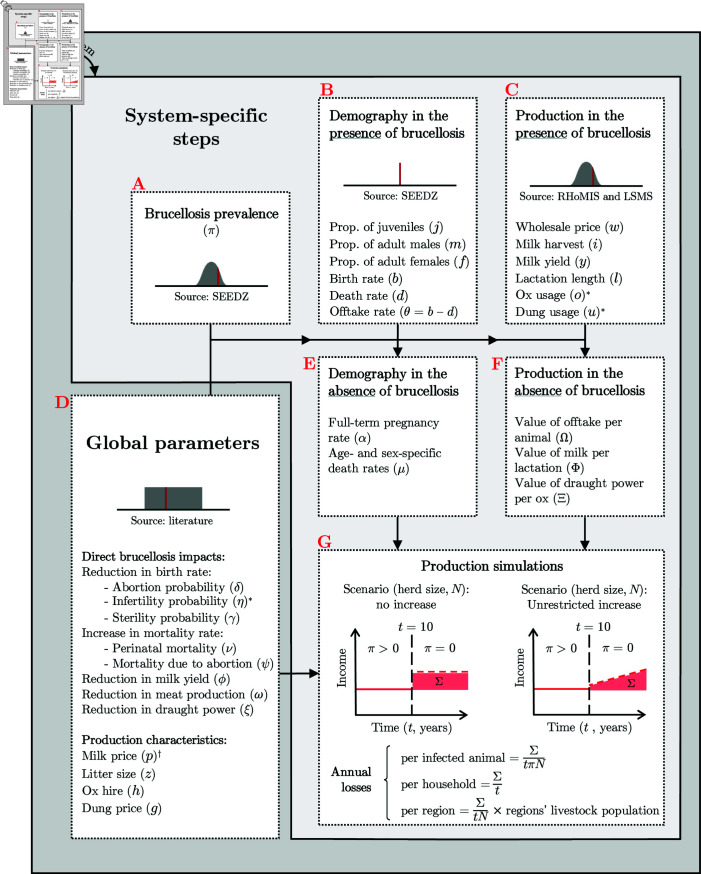
Workflow used to estimate losses attributable to brucellosis in livestock in northern and central Tanzania. Fixed parameter values are represented with a vertical red line. Parameter values obtained from distributions fitted to data are represented with a vertical red line in a grey normal distribution. Parameter values obtained from uniform distributions are represented with a vertical red line in a grey horizontal rectangle. Boxes A to G represent the step-wise processes. In the panel for step G (the model), the red shaded areas indicate the losses estimated in one iteration of the simulation (*∑*), under the two model scenarios considered: no increase (left) and unrestricted increase (right). ^*^ Parameters that entered the model as fixed values. ^*†*^ The values of milk price (p) were obtained from LSMS and RHoMIS and entered the model as distributions fitted to data.

The workflow was divided into two main layers (global and system-specific parameters) and seven interrelated steps (from A to G), as shown in Fig 1. Steps A to F consisted of processes that gathered and selected parameter values through several mechanisms: i) probabilistic distributions (system-specific distributions fitted to survey data - steps A and C; or non-system-specific, literature-based, uniform distributions - step D); and, ii) fixed values (system-specific demography - step B). Step A applied a Bayesian latent-class analysis (LCA) model to derive individual animal-level brucellosis prevalence estimates for pastoral and non-pastoral households in the SEEDZ dataset, as described by Bodenham et al. (2021) [15]. In this process, a simplifying assumption was made: an animal with serological evidence of exposure to *Brucella* spp. was considered ‘an infected animal’. Steps B and C gathered production-system-specific demographic and production values in the presence of brucellosis. Step D gathered from the literature global parameter values (same values across production systems) on direct brucellosis impacts and some production characteristics. The parameter values gathered and selected in steps A to D were used to estimate demography and production characteristics that would be expected in the absence of brucellosis (steps E and F). These steps were then followed by the modelling of herd production (simulations) in the presence and in the absence of brucellosis to estimate the losses attributable to the disease (step G). Losses were estimated per infected animal, per household, and per region. Losses attributable to each abortion were also estimated by linear regression using model outputs, i.e., losses per household, and abortions per herd, as regression variables. All monetary values were discounted over time at a rate of 3%. This means that the income from livestock is valued over time as a (discounted) present value. The discount rate of 3% aligns with the value used in a similar application in the study area [49]. The whole sequence of steps was repeated 50,000 times to fully explore the multidimensional parameter space. Each of these steps is described in detail in Supporting information (S3 File).

The model herd size (*N*) in each system, i.e., the initial number of animals of each species per household, was given by the median number of animals observed per household in each system in the SEEDZ survey. The expected consequences of the hypothetical, sudden elimination of brucellosis from the system on the herd size after ten years (simulation time horizon, *t* =  10 years) were modelled through two different model scenarios: i) ‘no increase in herd size’: all excess stock due to brucellosis elimination was valued as offtake (sold, slaughtered, or given away as gifts); and, ii) ‘unrestricted increase in herd size’. Unless otherwise stated, results refer to the first model scenario (‘no increase in herd size’; reference scenario) as this is likely the most appropriate to represent how households would manage herd size in the event of brucellosis elimination.

### Statistical analyses

General descriptive statistics were calculated for each of the main model outputs: i) losses per infected animal; ii) losses per household; and, iii) losses per region. Pearson’s Chi-squared test was used to assess differences between production systems in (i) demographic attributes of animal populations (e.g., proportion of animals in each age-sex group) and (ii) the proportion of households harvesting milk [50]. The herd sizes, the productivity of livestock (e.g., milk yield) as well as the estimated production losses per infected animal and per household were compared between production systems using the Wilcoxon rank-sum test [50]. The significance level was set at 5%. These comparative analyses were also carried out visually, through the inspection of violin and bar plots, which were made with the ‘ggpubr’ and ‘ggplot2’ packages, in R [51,52]. The influence of the model parameter values on the estimated production losses was assessed through elasticity analyses [53]. Elasticity analyses estimated the percentage change in production losses (per infected animal and household) that resulted from a percentage change in parameter values. These analyses were performed separately for the two model scenarios: ‘no increase in herd size’ and ‘unrestricted increase in herd size’.

Elasticity was calculated using a log-log function, as follows [54]:


$$\begin{array}{ccc} ln\left(y\right)=I+E\times ln\left(x\right) & & \end{array}$$ (1)

where *y* is the production loss per infected animal in each species, *I* is the regression intercept, *E* is the elasticity (percentage change in production loss that results from a percentage change in the parameter value), and *x* is the input parameter.

## Results

### Household classification by production system

**Fig 2:**
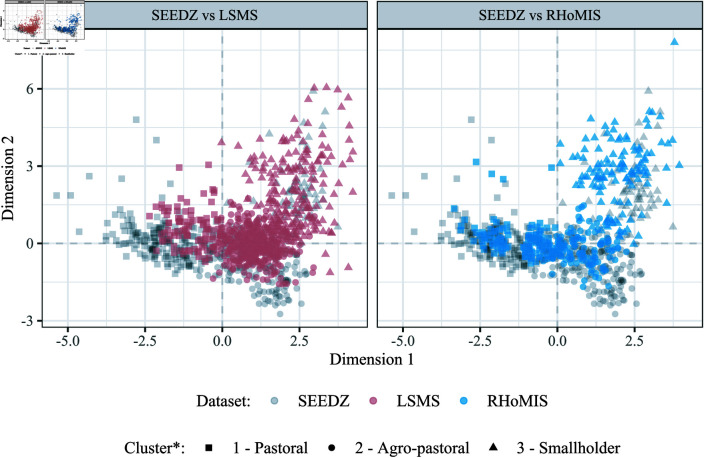
Individual scores of LSMS and RHoMIS households in shaped and coloured dots, as compared to SEEDZ’s (grey squares, as per de Glanville et al. (2020) [16]), on the first and second factors of the multiple factor analysis, and membership of each household to a cluster. Results obtained with the best-performing model: a model with all SEEDZ variables, except those that were missing in LSMS, integrated ‘one by one’ to the whole SEEDZ dataset, and with partition of the hierarchical tree constrained to three clusters. SEEDZ refers to the Social, Environmental, and Economic Drivers of Zoonotic disease project. LSMS refers to the Living Standards Measurement Study. RHoMIS refers to the Rural Household Multiple Indicator Survey.

The percentage of households classified as pastoral with the best-performing model was 42.8% (173 out of 404), 12.9% (96 out of 746), and 29.4% (115 out of 391) in SEEDZ, LSMS, and RHoMIS, respectively. The individual scores of LSMS and RHoMIS households on the first and second factors from the multiple factor analysis followed broadly the distribution of SEEDZ households scores of M0. This result is shown in Fig 2, where three distinct clusters are visible in LSMS and RHoMIS, overlapping those in SEEDZ: i) cluster one, predominately negative scores on dimension 1, consistent with pastoral households; ii) cluster two, predominately positive scores on dimension 1, consistent with agro-pastoral households; and, iii) cluster three, predominately positive scores on dimensions 1 and 2, consistent with smallholder households [16].

### Livestock brucellosis prevalence

**Fig 3:**
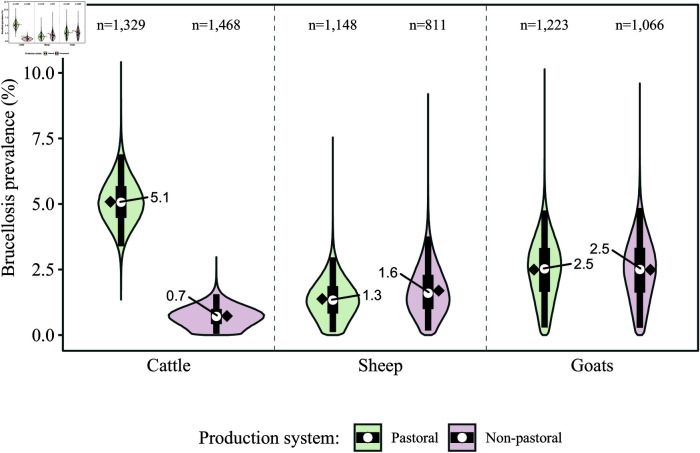
Latent-class analysis-derived brucellosis prevalence estimates by production system (pastoral and non-pastoral) and species (cattle, sheep, and goats). White dots indicate the median, black diamonds the mean, black boxes the 50% credible interval, and black lines the 95% credible interval. ‘n’ indicates the number of animals sampled and tested for brucellosis with both the Rose Bengal test (RBT) and competitive enzyme-linked immunosorbent assay (cELISA).

The estimates of individual-level brucellosis prevalence by species and production system, obtained with the latent-class analysis model, are shown in Fig 3. The highest prevalence estimates were in pastoral cattle (median of 5.1%), which were over seven times more likely to be infected than non-pastoral cattle (median of 0.7%). In small ruminants, the prevalence estimates were similar across production systems, and slightly higher in goats (median of 2.5% in both systems) compared to sheep (median of 1.3 and 1.6% in pastoral and non-pastoral systems, respectively).

### Production losses attributable to livestock brucellosis

**Fig 4:**
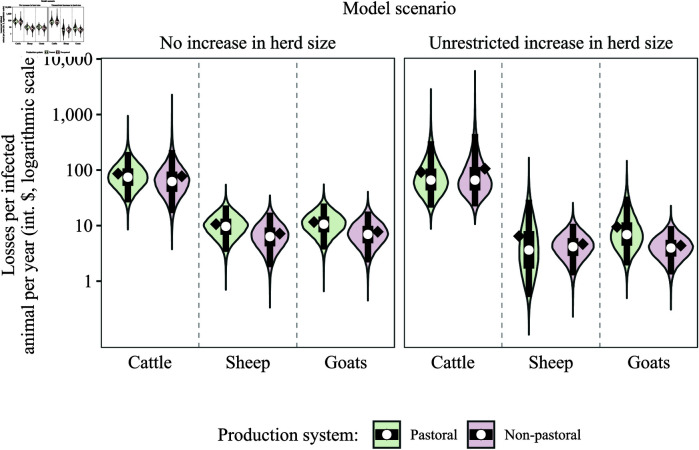
Estimated production losses attributable to brucellosis per infected animal per year, by production system (pastoral and non-pastoral), species (cattle, sheep, and goats), and model scenario (‘no increase in herd size’ and ‘unrestricted increase in herd size’). White dots indicate the median, black diamonds the mean, black boxes the inter-quartile range (50% UI), and black lines the 2.5 and 97.5 percentiles (95% UI). Uncertainty intervals were obtained from Monte Carlo simulation (50,000 iterations). The values in the y-axis are on the logarithmic scale. Int. $: International dollars.

The estimated production losses per infected animal, by species, production system, and model scenario, are shown in Fig 4. Under the model scenario of ‘no increase in herd size’ (left panel in Fig 4), the median (95% uncertainty interval, UI) annual losses attributable to brucellosis in cattle, sheep, and goats, per infected animal, were 74.4 (26.2-211.7), 9.7 (3.4-23.1), and 10.6 (3.7-25.0) international dollars (int. $) in the pastoral system, and 62.3 (16.8-228.6), 6.3 (1.8-17.1), and 7.0 (2.2-17.9) int. $ in the non-pastoral system, respectively. In cattle, the estimated production losses were approximately seven to ten times as high as those estimated in sheep and goats. For all species, greater losses were estimated in the pastoral system, compared to the non-pastoral system. Additionally, within the same system, greater losses per infected animal were estimated in goats, compared to sheep.

Under the model scenario of ‘unrestricted increase in herd size’ (right panel in Fig 4), the median (95% UI) annual losses attributable to brucellosis in cattle, sheep, and goats, per infected animal, were 66.0 (21.1–329.2), 3.6 (0.5–29.2), and 6.9 (1.9–33.2) int. $ in the pastoral system, and 65.9 (22.3–445.0), 4.2 (1.3–10.8), and 4.0 (1.3–9.8) int. $ in the non-pastoral system, respectively. The difference in losses between cattle and the other species was slightly greater in the model scenario of ‘unrestricted increase in herd size’, compared to the scenario of ‘no increase in herd size’.

**Fig 5:**
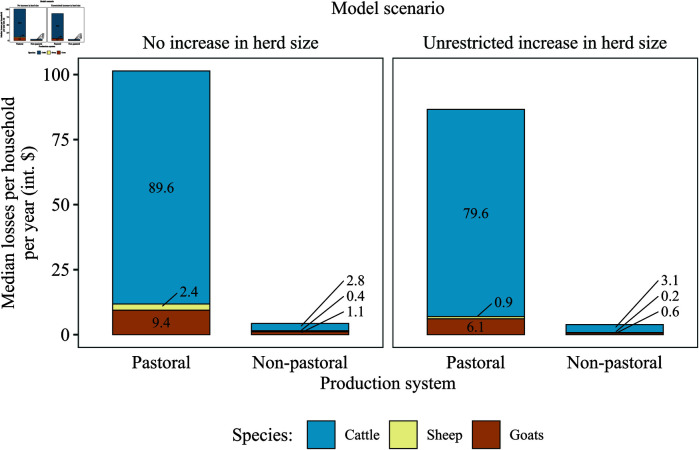
Estimated production losses attributable to brucellosis per household per year, by production system (pastoral and non-pastoral), species (cattle, sheep, and goats), and model scenario (‘no increase in herd size’ and ‘unrestricted increase in herd size’). The numbers within each bar or connected to it with a line segment indicate the median household-level losses attributable to brucellosis in each species. Estimates of household-level losses assume a herd size of 24 cattle, 20 sheep, and 38 goats in the pastoral system, and seven cattle, four sheep, and seven goats in the non-pastoral system. Int. $: International dollars.

The estimated production losses per household, by species, production system, and model scenario, are shown in Fig 5. The household-level losses attributable to brucellosis in the pastoral system (101.4 int. $ per year) were equivalent to 4.4% (95% UI: 2.1–8.8) of the median annual, livestock-derived, household income expected in the absence of the disease (approximately 2,265 int. $; model scenario of ‘no increase in herd size’). In the non-pastoral system, the household-level losses attributable to brucellosis (4.3 int. $ per year) were equivalent to 0.6% (95% UI: 0.2–1.6) (near eight-fold lower than in the pastoral system) of the median annual, livestock-derived, household income expected in the absence of the disease (approximately 755 int. $; model scenario of ‘no increase in herd size’). Cattle contributed the most to household-level losses in both production systems and model scenarios. Sheep and goats jointly contributed around one-tenth and less than one-fourth of the household-level losses in pastoral and non-pastoral systems, respectively. In cattle, sheep, and goats, each abortion represented a median loss of 1,054.6 (1,034.6–1,074.6), 142.8 (141.6–144.0), and 134.5 (133.5–135.6) int. $ in the pastoral system, and 1,599.8 (1,574.9–1,624.7), 133.7 (132.5–134.9), and 106.8 (105.9–107.7) int. $ in the non-pastoral system, respectively.

**Fig 6:**
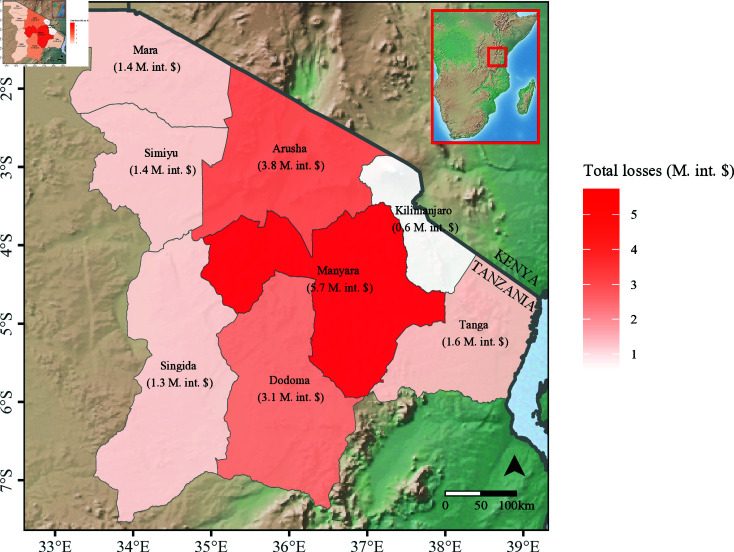
Choropleth map of the median losses attributable to brucellosis in the study area, northern and central Tanzania, per year, by region. The estimates of production losses were obtained from the reference model scenario (‘no increase in herd size’). Solid lines delineate regional and national borders. M. int. $: million international dollars. The inset at the upper right corner shows central and southeastern parts of Africa, with the study area highlighted in red. This figure was made in R [46] with packages ‘ggplot2’, ‘raster’, ‘sf’, and ‘rgdal’ [44,45,51,55,56]. The polygon shapefiles were obtained from ‘GADM’, the Database of Global Administrative Areas (https://gadm.org/). The base map was obtained from ‘Natural Earth’: ‘Cross Blended Hypso with Shaded Relief and Water’ raster, version 2.0.0 (www.naturalearthdata.com).

The annual, region-level losses attributable to brucellosis in northern and central Tanzania are shown in Fig 6. Manyara was the region with the highest estimated production losses per year (5.7 million (M.) int. $), followed by Arusha (3.8 M. int. $) and Dodoma (3.1 M. int. $). These three regions were those with the largest population of livestock kept by pastoral households, as indicated in Table F in Supporting information (S3 File). Kilimanjaro was the region with the lowest estimated production losses, at 0.6 M. int. $ per year. In this region, livestock were kept predominantly by non-pastoral households.

### Determinants of production losses attributable to brucellosis

**Fig 7:**
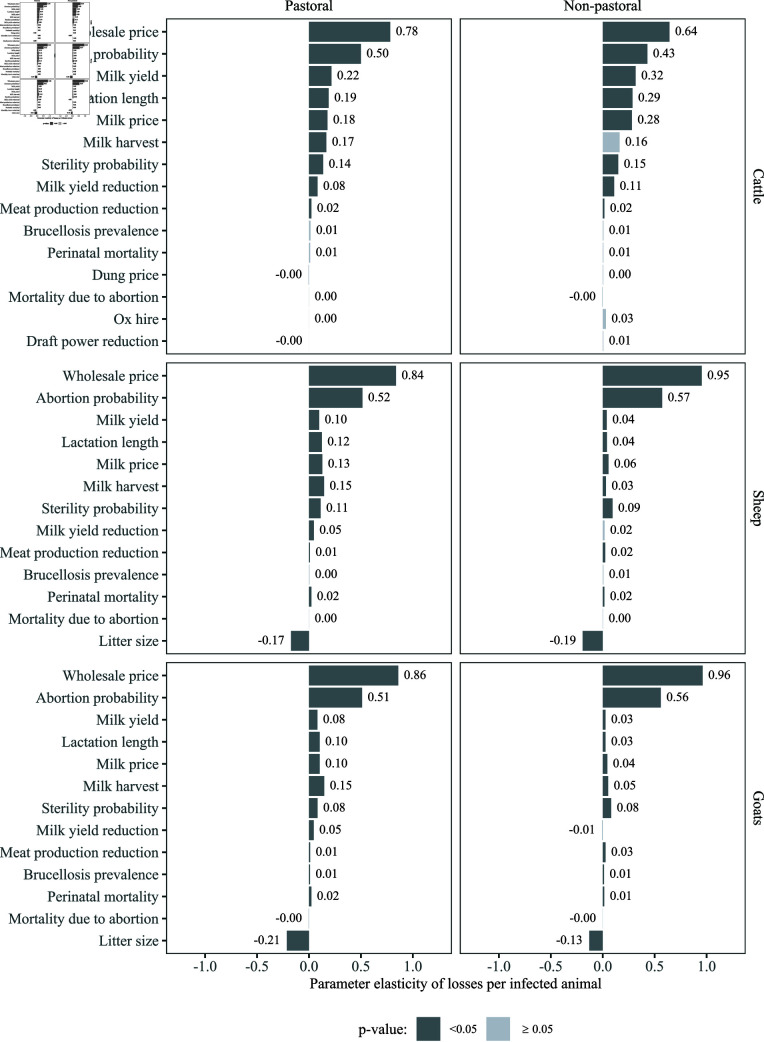
Tornado plot showing the parameter elasticity of production losses per infected animal (percentage change in production losses per infected animal per year when parameter values change by 1%), by production system (pastoral and non-pastoral) and species (cattle, sheep, and goats) (50,000 model iterations, scenario of ‘no increase in herd size’). The colours of the bars indicate the significance of the parameter elasticity estimate (dark and light grey indicate p-value  <  0.05 and p-value  ≥  0.05, respectively).

The results of the elasticity analyses of production losses per infected animal per year, under the model scenario of ‘no increase in herd size’, are shown in Fig 7. The parameters for which variation affected the estimated production losses per infected animal the most were the wholesale price and the abortion probability. This was consistent for all species (cattle, sheep, and goats) and both production systems (pastoral and non-pastoral). The next most influential variables were related to milk value (milk yield, lactation length, milk price, and milk harvest) in cattle and litter size in sheep and goats. Further results from elasticity analyses are shown in Supporting information (at household level under the model scenario of ‘no increase in herd size’, and at the individual and household levels under the scenario of ‘unrestricted increase in herd size’; Figs A-C; S4 File).

## Discussion

This study developed an integrated epidemiological-economic modelling framework to estimate the production losses attributable to livestock brucellosis in endemic settings. This framework was applied in the context of northern and central Tanzania, in pastoral and non-pastoral systems, which are known to differ considerably in their livestock management practices and brucellosis prevalence [15,16].

For contextualisation, it is useful to compare the estimated production losses from brucellosis with the value of livestock production in the study area. The median estimated production losses per infected animal per year were equivalent to approximately 20% of the median wholesale price in the pastoral system, for all species, and lower (closer to 10%) in the non-pastoral system (model scenario of ‘no increase in herd size’). Alternatively, these losses per infected animal were equivalent to 39%, 15%, and 16% of the value of each lactation (milk yield times lactation length times milk price) in cattle, sheep, and goats in the pastoral system, respectively, and approximately 10% of the median value of each lactation for all species in the non-pastoral system. For wholesale prices, milk yield, and milk prices, see Supporting information (steps C and D; S3 File).

The median losses per household in the pastoral system were over 20 times as high as those in the non-pastoral system in both model scenarios. The impact of brucellosis in livestock is then inextricably linked with the context in which livestock are raised. The production system plays a clear role in determining the losses attributable to brucellosis. The higher prevalence of the disease, at least in cattle, as well as the greater dependency on livestock and livestock products for their livelihoods, as measured by livestock and livestock product harvest rates (e.g., number of animals slaughtered and volume of milk harvested per year), results in significantly greater losses in pastoral households, compared to those in the non-pastoral system. These results indicate the importance of deriving context-specific estimates in future assessments of livestock brucellosis impacts.

The total estimated production loss attributable to brucellosis per year in the study area was 19.0 million int. $ (the sum of the median estimated production losses in the eight regions: Arusha, Manyara, Kilimanjaro, Tanga, Dodoma, Singida, Simiyu, and Mara), equivalent to 0.71 int. $ per animal kept (including all species: cattle, sheep, or goats). Given the differences in household-level losses between production systems and the relative distribution of livestock by regions, considerable heterogeneity in regional losses was identified. A near ten-fold difference was estimated between the regions with the highest and the lowest estimated production losses (Manyara, with 5.7 million int. $ per year, and Kilimanjaro, with 0.6 million int. $ per year). The spatial heterogeneity in livestock production losses may be relevant for designing and prioritising potentially targeted control interventions.

Our estimates of production losses attributable to each brucellosis-induced abortion in cattle were 1,054.6 (1,034.6–1,074.6) int. $ in the pastoral system and 1,599.8 (1,574.9–1,624.7) int. $ in the non-pastoral system. These values were in line with the range of estimated *ex-post* costs per abortion in northern Tanzania [49]. According to a recent study, the gross abortion losses (assuming milk is used by households after abortion) were estimated at Tanzanian Shilling (TZS) values equivalent to 464.8 int. $ and 1,439.3 int. $ for local and non-local breeds of cattle, respectively (using a World Bank conversion factor of 743.95 TZS per int. $, in 2018, available at https://data.worldbank.org) [49]. This relative between-study consistency in estimated production losses for each brucellosis-induced abortion event also applies to small ruminants.

The livestock production losses attributable to brucellosis estimated in this study were lower than those reported elsewhere. The median losses per infected animal in the reference model scenario were between 62.3 and 74.4 int. $ per year in cattle (non-pastoral and pastoral systems, respectively) and below or equal to 10.6 int. $ per year in sheep and goats in both pastoral and non-pastoral systems. Elsewhere, the losses per infected animal have been estimated at over 100 int. $ in cattle (India, Brazil, Colombia, Uganda, Turkey, and Sudan) and over 20 int. $ in sheep and goats (India, Iraq, and Malaysia) [17,18,24,25,27–30,57–59] (using the country of each study, their local currency, year of data collection and the corresponding World Bank conversion rates at https://data.worldbank.org).

There are many possible explanations for the relatively lower losses estimated in this study. First, there is significant heterogeneity in methods used elsewhere, which hamper comparisons between study systems [21]. In contrast to the present study, other studies estimating the impact of livestock brucellosis often value specific impacts of the disease (e.g., temporary infertility or sterility) at the cost, at least partially, of the replacement animal (e.g., the price of a fertile adult animal) [17,24]. This practice overestimates the loss caused by brucellosis because the offtake value of an infected animal is often not fully depreciated by the occurrence of sterility [60]. Another recurring practice is the use of fixed market prices collected at national or sub-national levels to value livestock and livestock products [17,24]. The use of fixed market prices to estimate losses attributable to brucellosis has two disadvantages: i) market prices may be time- and context-specific, varying with factors such as supply and demand, and may not reflect the value that households put on their animals [61]; and, ii) as shown in the present study, the value of livestock and livestock products is the most important determinant of the losses per infected animal. Studies that use fixed livestock and livestock product market prices may fail to capture the likely substantial variation and uncertainty in estimated production losses, which can influence the accuracy of central-point estimates. The central-point estimate most commonly reported is the mean, which tends to be greater than the median when the distribution of production losses is skewed to the right [50]. The present study used mostly distributions of actual prices reported by households based on sales instead of fixed market prices.

Second, a potential source of differences in results between studies is the way authors value the foregone losses. Given the long-term effects of brucellosis on reproduction and demography, foregone losses, which include, for instance, the milk loss from unborn female offspring, are a non-negligible part of the overall impact of the disease on livestock productivity [24]. Foregone losses are also intrinsically challenging to estimate because they are (i) dependent on herd management decisions, (ii) time-bounded, and (iii) difficult to separate from current production losses (e.g., due to reduced growth rate). For these reasons, modelling approaches such as the one described in the present study that (i) take into account the herd-growth dynamics, (ii) simulate livestock production for a period of time in the presence and in the absence of the disease, and (iii) estimate current and foregone losses simultaneously may be good alternatives to previously used models. Additionally, many studies that estimated the losses attributable to brucellosis do not discount foregone losses over time, which overestimates these losses, as viewed from the present [17,18,24,28].

The model framework proposed in this study explores the consequences of different herd size management decisions through two scenarios over the ten-year time horizon considered: i) ‘no increase in herd size’ and ii) ‘unrestricted increase in herd size’. Varying the time horizon would likely result in different estimated production losses. The effect of time horizon on estimated production losses varies between production systems, as livestock are managed and valued differently between pastoral and non-pastoral households. Our results demonstrate the need for future studies on the losses attributable to livestock brucellosis to account for the effect of herd size management decisions and different time horizons.

Third, this study aimed to estimate losses attributable to brucellosis, as defined by McInerney, Howe, and Schepers (1992) [19], i.e., the production potential that is not realised over time due to the presence of the disease. This definition of ‘losses’ is in line with the conceptual approach proposed in the recently published GBADs Technical Guide for animal disease burden assessment and excludes prevention and treatment expenditures and other averting expenditures due to the presence of the disease [20]. However, some studies have included these costs (e.g., cost of veterinary assistance, or cost of repeat breeding) in the estimation of ‘losses’ [17,18]. Other studies have used a similar definition of losses, but the range of livestock products considered differed from the ones in this study. For instance, in the present study, for sheep and goats, only the losses in offtake and milk values were estimated because these are the main, if not the only, sources of income from these species to households in the study area. In other studies, for the same livestock species, losses from other outputs (e.g., wool) were also considered [24].

The production losses attributable to livestock brucellosis estimated in this study in northern and central Tanzania are novel and can help inform policy decisions regarding disease control. The results of this study indicate the need for targeted, context-specific disease control strategies that capitalise on the knowledge of the heterogeneous distribution of brucellosis impacts by livestock species, production systems, and geographical areas. Brucellosis control interventions that target livestock species and systems in which losses are greater are likely to yield higher marginal benefits than non-targeted strategies [62]. Examples of targeted interventions that could be cost-beneficial include vaccination of cattle and education campaigns on disease prevention and control in pastoral communities. Further research is needed to evaluate the societal benefits of such interventions, including to human health. This wider evaluation could also explore cost-sharing scenarios that would enable disease control costs to be funded by sectors of society in proportion to benefits [63].

Some limitations of this study need to be highlighted. One of the main limitations was the uncertain representativeness of prevalence data. The SEEDZ survey tested 7,045 livestock (cattle, sheep, and goats) from 404 households in Arusha and Manyara. SEEDZ did not sample animals from other regions. The degree to which the prevalence data in SEEDZ were representative of the other regions in the study area is unknown. The study by Bodenham et al. (2021) [15] applied the same latent-class model to analyse prevalence data from SEEDZ and another survey (BacZoo) delivered in Kilimanjaro. Prevalence data from BacZoo were not included in the present study because the survey excluded many variables needed for the clustering algorithm. The prevalence estimates reported by Bodenham et al. (2021) [15] in the pastoral system were higher than in the current study (6.3% vs 5.1% in cattle; 3.3% vs 1.3% in sheep; and, 5.1% vs 2.5% in goats). Other epidemiological studies carried out in northern Tanzania or elsewhere in the country often report a higher prevalence of livestock brucellosis than estimated in the present study [64–78]. Therefore, our study may have underestimated brucellosis-related losses at the individual and population level.

The model framework used in this study minimised the likelihood of overestimating production losses in the presence of comorbidities, a common problem in disease impact estimation studies [79]. The demographic and production parameters in the model, both in the presence and hypothetical absence of brucellosis, are calculated given data on disease prevalence and direct pathological impacts (e.g., probabilities of abortion or perinatal mortality in infected animals). Whilst this approach minimises the risk of overestimating production losses, the estimates generated are based on the source data selected and may be affected by imperfections in these data. Besides the uncertain representativeness of prevalence data mentioned above, there is currently limited quantitative evidence on the direct pathological impacts of brucellosis, particularly in small ruminants [8]. Methods employed for this study attempted to account for uncertainty in available quantitative evidence and the elasticity analyses performed explicitly assess the implications of these uncertainties for our model outcomes. More research is needed to address this knowledge gap and help inform further brucellosis impact estimation studies.

The small number of households providing information on production characteristics, particularly of sheep and goats, is another limitation of this study. For instance, the wholesale prices of sheep and goats in the pastoral system were obtained from 20 and 41 households only, respectively (Table D in Supporting information; S3 File). It could be argued that this small sample of households may have provided a biased estimate of wholesale prices. The median prices reported by these households (57 int. $ for both sheep and goats) were lower than reported nationwide in the latest livestock sector analysis led by the Tanzanian Government: between 80 and 119 int. $ for adult male sheep and goats [80] (using the World Bank conversion rates for Tanzania in 2017 available at https://data.worldbank.org/). However, the median wholesale price of goats in the non-pastoral system in the present study (54 int. $), which was based on answers from many more households (n=181), was also lower than in the livestock sector analysis report. This indicates that the wholesale prices used in the present study are likely specific to the study area, rather than potentially biased.

Finally, the cross-sectional nature of the survey that provided data on demographic characteristics of the livestock population (SEEDZ) is another potential limitation of the present study. It is unclear whether the year in which the SEEDZ survey was delivered (2016) accurately represented the demographic characteristics of the same livestock population in other years. If there were abnormal birth and death rates in 2016, the losses estimated in 10-year simulations could be biased. Note that the offtake rate (*θ* ) ( *b* ) ( *d*) rates. If 2016 was a ‘good’ year, with higher birth rates and lower death rates than usual, the losses reported here are likely overestimated. In contrast, if 2016 was a ‘bad’ year, with lower birth rates and higher death rates than usual, the losses reported here are likely underestimated. The latter (2016 being a ‘bad’ year) is plausible because higher offtake rates ( >  10%) have been reported in other years and for all species in Tanzania [80].

## Conclusion

Brucellosis causes a significant economic impact on the livestock sector of the study regions in northern and central Tanzania, with estimated annual losses amounting to approximately 19 million int. $. The distribution of estimated production losses across households and geographical areas is highly heterogeneous. A key determinant of this heterogeneity is the production system, with losses occurring predominantly in pastoral households and in regions where these households are concentrated (Arusha and Manyara). Future research projects aimed at estimating the impact of livestock brucellosis in a similar context should then be designed taking into account the differences between production systems.

A new model framework for estimating the losses attributable to brucellosis was proposed in this study. This framework is novel for its ability to capture foregone losses attributable to low reproductive performance. This ability makes the framework suitable for application in the context of other diseases characterised by high reproductive morbidity and low mortality, in Tanzania and similar settings. The outcomes of this study may be helpful to inform future cost-benefit analyses of potential brucellosis control interventions.

## Supporting information

S1 File Household surveys and environmental data. (PDF)

S2 File Description of the harmonised household classification models. (PDF)

S3 File Estimation of production losses attributable to livestock brucellosis by production system. (PDF)

S4 File Determinants of production losses attributable to brucellosis. (PDF)

## Acknowledgments

The authors thank all individuals and organisations that contributed data used in this study and provided clearance for this research.
