# Supplementary material for: Livestock production losses attributable to brucellosis in northern and central Tanzania: Application of an epidemiological-economic modelling framework
Source: PLoS Negl Trop Dis. 2025 Feb 14;19(2):e0012814. doi: 10.1371/journal.pntd.0012814 (PMC11828364; doi:10.1371/journal.pntd.0012814)
Supplement: S1 File — (PDF) [file pntd.0012814.s001.pdf]

Livestock production losses attributable to brucellosis in northern and central Tanzania: application of an epidemiological-economic modelling framework

Ângelo J. F. Mendes[1*], Daniel T. Haydon[1], William A. de Glanville[1], Rebecca F. Bodenham[1], AbdulHamid S. Lukambagire[2], Paul C. D. Johnson[1], Gabriel M. Shirima[3], Sarah Cleaveland[1], Emma McIntosh[4], Nick Hanley[1], Jo E. B. Halliday[1]

1 School of Biodiversity, One Health and Veterinary Medicine, College of Medical, Veterinary and Life Sciences, University of Glasgow, Glasgow, United Kingdom
2 Kilimanjaro Clinical Research Institute, Kilimanjaro Christian Medical University College, Moshi, Tanzania
3 School of Life Sciences and Bioengineering, The Nelson Mandela African Institution of Science and Technology, Arusha, Tanzania
4 School of Health and Wellbeing, College of Medical, Veterinary and Life Sciences, University of Glasgow, Glasgow, United Kingdom

* a.mendes.1@research.gla.ac.uk

Supporting information

S1 File. Household surveys and environmental data

Table A: Timing, spatial extent, and number of households included in each survey selected for data harmonisation and identification of livestock production systems.

	Survey*				
	SEEDZ	LSMS3	LSMS4	RHoMIS1	RHoMIS2
Year	2016	2012-2013	2014-2015	2017	2018
Spatial extent of the full survey in Tanzania (regions)	Arusha, Manyara	Nationwide	Nationwide	Nationwide	Dodoma, Iringa, Tabora, Zanzibar
Households in the full survey (n)	404	5,010	3,352	994	841
Households surveyed in the study area (n)◇	404	935	712	302	206
Livestock-keeping households surveyed in the study area (n)◇,†	404	427	319‡	302	89

* SEEDZ refers to the Social, Environmental, and Economic Drivers of Zoonotic disease project. LSMS3 and LSMS4 refer to the third and fourth waves of the Living Standards Measurement Study, respectively. RHoMIS1 and RHoMIS2 refer to the Rural Household Multiple Indicator Survey applications led by the International Livestock Research Institute and the International Centre for Research in Agroforestry, respectively. ◇ Confirmed by GPS location, i.e., located in one of the following regions of northern and central Tanzania: Arusha, Manyara, Kilimanjaro, Tanga, Dodoma, Singida, Simiyu, and Mara. † Number of households from each survey that were included in this study. ‡ One household with the same identification number as in wave 3 was excluded.

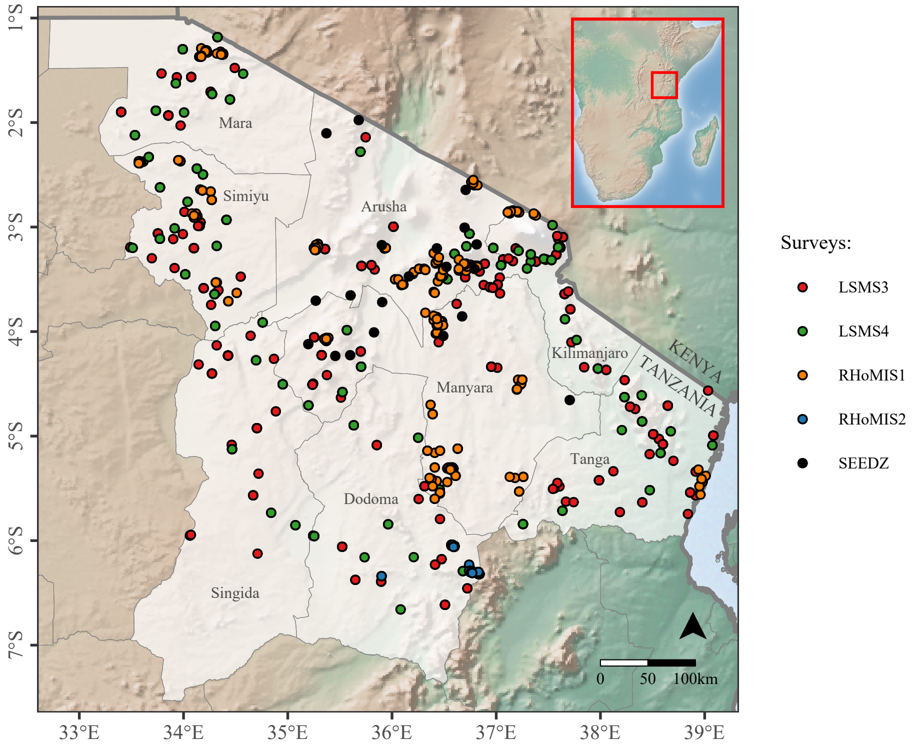

Figure A: Spatial distribution of households from each survey included in this study. SEEDZ refers to the Social, Environmental, and Economic Drivers of Zoonotic disease project (black dots represent villages visited). LSMS3 and LSMS4 refer to the third and fourth waves of the Living Standards Measurement Study, respectively. RHoMIS1 and RHoMIS2 refer to the Rural Household Multiple Indicator Survey applications led by the International Livestock Research Institute and the International Centre for Research in Agroforestry, respectively. Coloured dots indicate households' approximate geolocation, which has been modified to preserve confidentiality. White-filled translucent polygons indicate the study area. Solid lines delineate regional and national borders. The inset at the upper right corner shows the central and southeastern parts of Africa, with the study area highlighted in red. This figure was made in R [1] with packages 'ggplot2', 'raster', 'sf', and 'rgdal' [2–6]. The polygon shapefiles were obtained from 'GADM', the Database of Global Administrative Areas (`https://gadm.org/`). The base map was obtained from 'Natural Earth': 'Cross Blended Hypso with Shaded Relief and Water' raster, version 2.0.0 (`www.naturalearthdata.com`).

Table B: Description of environmental variables used in the household classification models and corresponding data sources. Defined spatial extent: northern and central Tanzania (Arusha, Manyara, Kilimanjaro, Tanga, Dodoma, Singida, Simiyu, and Mara).

Environmental variable	Description	Data source
Annual precipitation	Annual precipitation in mm	www.worldclim.org/bioclim
Average temperature	Annual average mean temperature in ^{o}C	www.worldclim.org/bioclim
Average vegetation cover	Annual average enhanced vegetation index (EVI)	https://lpdaac.usgs.gov/products/mod13q1v006
Cattle density	Number of cattle per km^2	www.fao.org/ag/againfo/resources/en/glw/GLW_dens.html
Chicken density	Number of chicken per km^2	www.fao.org/ag/againfo/resources/en/glw/GLW_dens.html
Cropland cover	Proportion of an area within 1 km radius classified as cropland*	https://landsat.gsfc.nasa.gov/data◇
Distance to main road	Euclidean distance to the nearest trunk road (in km) using the Distance Matrix tool in QGIS	www.fao.org/geonetwork/srv/en/main.home
Forest cover	Proportion of an area within 1 km radius classified as forest*	https://landsat.gsfc.nasa.gov/data◇
Goats density	Number of goats per km^2	www.fao.org/ag/againfo/resources/en/glw/GLW_dens.html
Grassland cover	Proportion of an area within 1 km radius classified as grassland*	https://landsat.gsfc.nasa.gov/data◇
Human population density	Human population per km^2 in 2016	www.worldpop.org.uk
Maximum slope	Maximum slope (in degrees) within 1 km radius†	https://lta.cr.usgs.gov/srtmgl3.html
Pigs density	Number of pigs per km^2	www.fao.org/ag/againfo/resources/en/glw/GLW_dens.html
Sheep density	Number of sheep per km^2	www.fao.org/ag/againfo/resources/en/glw/GLW_dens.html
Travel time to market centre	Travel time (in hours) to a town of greater than 20,000 people	http://dx.doi.org/10.7910/DVN/YKDWJD
Village area	Area of the village (in decimal degrees)	www.nbs.go.tz

* For the candidate datasets, the proportion of the buffer area classified as each land cover type (cropland, grassland, and forest) was used. ◇ For the candidate datasets, the land cover data were downloaded from https://lpdaac.usgs.gov/products/mcd12q1v006. † For the candidate datasets, SRTM (Shuttle Radar Topography Mission) data were downloaded using the SRTM-Downloader plugin [7] in QGIS [8], and the mean slope within the buffer was used.

References

1. R Core Team. R: A language and environment for statistical computing. R Foundation for Statistical Computing, Vienna, Austria. Version 3.6.2. 2019.

2. Wickham H. ggplot2: Elegant graphics for data analysis. Springer-Verlag New York; 2016.

3. Hijmans RJ. raster: Geographic Data Analysis and Modeling; 2023. Available from: `https://CRAN.R-project.org/package=raster`.

4. Pebesma E. Simple Features for R: standardized support for spatial vector data. Version 0.9-6. The R Journal. 2018;10(1):439–446. doi:10.32614/RJ-2018-009.

5. Pebesma E, Bivand R. Spatial Data Science: With Applications in R. Chapman and Hall/CRC; 2023.

6. Bivand R, Keitt T, Rowlingson B. rgdal: Bindings for the 'Geospatial' Data Abstraction Library; 2023. Available from: `https://CRAN.R-project.org/package=rgdal`.

7. Duester H. SRTM-Downloader (version 3.1.10). 2021.

8. QGIS org. QGIS Geographic Information System. QGIS Association. Version 3.10.6-A Coruña. 2020.
