# Supplementary material for: Livestock production losses attributable to brucellosis in northern and central Tanzania: Application of an epidemiological-economic modelling framework
Source: PLoS Negl Trop Dis. 2025 Feb 14;19(2):e0012814. doi: 10.1371/journal.pntd.0012814 (PMC11828364; doi:10.1371/journal.pntd.0012814)
Supplement: S2 File — (PDF) [file pntd.0012814.s002.pdf]

Livestock production losses attributable to brucellosis in northern and central Tanzania: application of an epidemiological-economic modelling framework

Ângelo J. F. Mendes[1*], Daniel T. Haydon[1], William A. de Glanville[1], Rebecca F. Bodenham[1], AbdulHamid S. Lukambagire[2], Paul C. D. Johnson[1], Gabriel M. Shirima[3], Sarah Cleaveland[1], Emma McIntosh[4], Nick Hanley[1], Jo E. B. Halliday[1]

1 School of Biodiversity, One Health and Veterinary Medicine, College of Medical, Veterinary and Life Sciences, University of Glasgow, Glasgow, United Kingdom
2 Kilimanjaro Clinical Research Institute, Kilimanjaro Christian Medical University College, Moshi, Tanzania
3 School of Life Sciences and Bioengineering, The Nelson Mandela African Institution of Science and Technology, Arusha, Tanzania
4 School of Health and Wellbeing, College of Medical, Veterinary and Life Sciences, University of Glasgow, Glasgow, United Kingdom

* a.mendes.1@research.gla.ac.uk

Supporting information

S2 File. Description of the harmonised household classification models

As performed in M0 by de Glanville et al. (2020) [1], all continuous variables were scaled in each of the classification models either through a Z-score or log transformation, depending on the distribution of the variable. All models were run with imputation of missing values using the regularised iterative multiple factor analysis (MFA) algorithm of the 'missMDA' package (version 1.17) [2] in R [3].

The first stage of the clustering algorithm consisted of dimension reduction using MFA. MFA is a procedure that simplifies and denoises the informative value of continuous and categorical variables that are organised in meaningful groups, e.g., in domains [3]. This stage was carried out with the 'MFA' function of the 'FactoMineR' package (version 2.3), in R. In the second stage, the low-dimensional data obtained from the MFA (factors) were used to identify clusters of households, i.e., households sharing similar characteristics, through hierarchical cluster analyses (HCA). A statistically significant v-test result (> 1.96, or p-value < 0.05; the v-test is a criterion with a Normal distribution [3]) was used as indicative of a difference in the mean value of the variable within a cluster, as compared to the mean value of the variable in the population. This second stage was carried out with the 'HCPC' (Hierarchical Clustering on Principal Components) function of the same package ('FactoMineR') in R. The partition of the hierarchical tree was automated, based on higher relative loss of inertia, which ensured the ability to identify a production system other than those obtained with M0. For those models with which the automated partition gave rise to more than three clusters (the number of clusters obtained with M0), a version of the model constrained to three clusters (named 'manual', distinct from 'auto', with automated partition) was also run. In any case, only factors associated with 'large' eigenvalues, based on the presence of a natural break on a scree plot, were included in the HCA.

Depending on the model run, between three and six factors associated with ‘large’ eigenvalues were included in the clustering procedure.

References

1. de Glanville WA, Davis A, Allan KJ, Buza J, Claxton JR, Crump JA, et al. Classification and characterisation of livestock production systems in northern Tanzania. PloS One. 2020;15(12):e0229478. doi:10.1371/journal.pone.0229478.

2. Josse J, Husson F. missMDA: A package for handling missing values in multivariate data analysis. Journal of Statistical Software. 2016;70(1):1–31. doi:10.18637/jss.v070.i01.

3. Lê S, Josse J, Husson F. FactoMineR: a package for multivariate analysis. Journal of Statistical Software. 2008;25(1):1–18. doi:10.18637/jss.v025.i01.
