# Supplementary material for: Livestock production losses attributable to brucellosis in northern and central Tanzania: Application of an epidemiological-economic modelling framework
Source: PLoS Negl Trop Dis. 2025 Feb 14;19(2):e0012814. doi: 10.1371/journal.pntd.0012814 (PMC11828364; doi:10.1371/journal.pntd.0012814)
Supplement: S3 File — (PDF) [file pntd.0012814.s003.pdf]

Livestock production losses attributable to brucellosis in northern and central Tanzania: application of an epidemiological-economic modelling framework

Ângelo J. F. Mendes[1*], Daniel T. Haydon[1], William A. de Glanville[1], Rebecca F. Bodenham[1], AbdulHamid S. Lukambagire[2], Paul C. D. Johnson[1], Gabriel M. Shirima[3], Sarah Cleaveland[1], Emma McIntosh[4], Nick Hanley[1], Jo E. B. Halliday[1]

1 School of Biodiversity, One Health and Veterinary Medicine, College of Medical, Veterinary and Life Sciences, University of Glasgow, Glasgow, United Kingdom
2 Kilimanjaro Clinical Research Institute, Kilimanjaro Christian Medical University College, Moshi, Tanzania
3 School of Life Sciences and Bioengineering, The Nelson Mandela African Institution of Science and Technology, Arusha, Tanzania
4 School of Health and Wellbeing, College of Medical, Veterinary and Life Sciences, University of Glasgow, Glasgow, United Kingdom

* a.mendes.1@research.gla.ac.uk

Supporting information

S3 File. Estimation of production losses attributable to livestock brucellosis by production system

Step A: Brucellosis prevalence

The first step of the analyses was the sampling of the probability distributions of brucellosis prevalence. These distributions of prevalence in cattle, sheep, and goat populations of pastoral and non-pastoral production systems were obtained from SEEDZ data through a Bayesian latent-class analysis (LCA) model. This model has been described in detail by Bodenham et al. (2021) [1], who used data from SEEDZ and another survey to estimate (i) the sensitivity and specificity of two serological tests (Rose Bengal test - RBT, and a competitive enzyme-linked immunosorbent assay - cELISA), and (ii) the prevalence of exposure to *Brucella* spp. in the same production systems: pastoral and non-pastoral. The classification of households by production system in Bodenham et al. (2021) [1] was based on agro-ecological definitions, in consultation with district-level government officials (i.e., District Veterinary Officers or District Livestock Officers). In the current study, instead, the household classification derived from the harmonised clustering algorithm with SEEDZ, LSMS, and RHoMIS households (S2 File) was used.

The LCA model used to estimate brucellosis prevalence in livestock was specified as per Bodenham et al. (2021) [1], but applied to the SEEDZ dataset only. This model can be briefly described in three parts:

1. Model framework and assumptions:

Separate models were set up for cattle, sheep, and goats. The overall model framework was adapted from the Hui-Walter model [1–4] using RBT and cELISA

outcome probabilities and prior information on (i) test performance (sensitivity and specificity), and (ii) prevalence of exposure to *Brucella* spp. in animals originating from the two production systems [1, 4, 5].

Two of the three core assumptions in the Hui-Walter model [1, 6] were considered not violated: i) the prevalence of exposure to *Brucella* spp. was different between production systems; and, ii) the diagnostic tests perform the same across production systems. A third assumption that the diagnostic tests were conditionally independent was conservatively considered not met because RBT and cELISA detect the presence of antibodies against *Brucella* spp. As a result, conditional dependence was added to the model through covariance parametrisation [1, 3, 7].

2. Prior distributions:

Priors following a beta distribution were specified in this model. Three sets of priors were used: i) the performance of RBT and cELISA (sensitivity and specificity); ii) the prevalence of exposure to *Brucella* spp.; and, iii) the covariance variables.

The priors of test performance, selected based on a literature search and sensitivity analysis of posterior distributions, are shown in Table A.

The priors for the prevalence of exposure to *Brucella* spp. were specified as vague uniform distributions (0, 0.49), enabling wide parameters space exploration.

The priors for the covariance variables were specified as uniform distributions with maximum and minimum limits dependent on the priors of test performance (as shown in Table A).

Table A: Beta distribution (dbeta) shape parameters (α, β) used to specify priors for sensitivity and specificity of the Rose-Bengal test (RBT) and competitive enzyme-linked immunosorbent assay (cELISA) for cattle, sheep, and goat models.

	Shape parameters (α, β) in each species model		
	Cattle	Sheep	Goats
RBT sensitivity	(8,1)	(4,1)	(4,1)
RBT specificity	(1,1)	(1,1)	(1,1)
cELISA sensitivity	(30,2)	(8,1)	(12,1)
cELISA specificity	(1,1)	(1,1)	(1,1)

The covariance variables (γ_{Se} and γ_{Sp}) were specified as uniform distributions with the maximum and minimum limits given below, as per Branscum et al. (2005) [5], Mazeri et al. (2016) [4], Dendukuri and Joseph (2001) [8], and Bodenham et al. (2021) [1]:

$$\begin{aligned}(Se_1 - 1)(1 - Se_2) \leq \gamma_{Se} \leq min(Se_1, Se_2) - Se_1 Se_2 \\ (Sp_1 - 1)(1 - Sp_2) \leq \gamma_{Sp} \leq min(Sp_1, Sp_2) - Sp_1 Sp_2\end{aligned} \tag{1}$$

where Se and Sp refer to sensitivity and specificity, respectively, and the subscripts $_1$ and $_2$ refer to the Rose-Bengal test (RBT) and a competitive enzyme-linked immunosorbent assay (cELISA), respectively.

3. Model implementation and diagnostics:

The model was implemented in R version 3.6.2 [9] with JAGS [10], using the rjags package [11]. Three Markov Chain Monte Carlo (MCMC) chains with different starting values were run for 250,000 iterations, discarding the first 50,000 as burn-in, and retaining every 100th iteration.

The Gelman and Rubin's diagnostic and the visual inspection of the Gelman-Rubin, density and trace plots were used to assess chain convergence, using the coda R package [12].

A sample of the posterior distribution of brucellosis prevalence for each species was used in each iteration of the production losses model. It was assumed that the prevalence of the disease was equally distributed across age and sex groups.

Step B: Demography in the presence of brucellosis

The age-sex distribution as well as the birth and death rates of livestock were obtained from SEEDZ data at the survey level, i.e., including all households in each production system, and were used in the model as fixed values. Adults were distinguished from juveniles in this survey based on the presence of permanent incisors. The parameter values in this step were obtained as follows:

- Proportion of juveniles (j), adult males (m), and adult females (f): The values of j, m, and f were obtained by dividing the number of animals in each age-sex group by the total number of animals kept at the time of the interview.
- Birth rate (b) and death rate (d): The values of b and d were obtained by dividing the total number of animals that were born and died in the 12 months preceding the interview, respectively, by the total number of animals kept at the time of the interview.
- Offtake rate (θ): the offtake rate in each year was given by the difference between the birth (b) and death (d) rates.

The number of animals kept by households surveyed in SEEDZ as well as the age-sex distribution of these animals and the corresponding birth, death, and offtake rates are shown in Table B. The median number of animals kept by pastoral households was 82

(24 cattle, 20 sheep and 38 goats), which was significantly greater than the number reported in the non-pastoral system (18 in total: seven cattle, four sheep, and seven goats) (p-value < 0.001).

Table B: Demography in the presence of brucellosis, by production system (pastoral and non-pastoral) and species (cattle, sheep, and goats), as calculated from the SEEDZ dataset.

	Pastoral			Non-pastoral		
	Cattle	Sheep	Goats	Cattle	Sheep	Goats
Total number of animals kept	10,108	8,697	9,934	2,334	1,839	2,679
Herd size per household*	24	20	38	7	4	7
Prop. of juveniles (j)	0.34	0.29	0.28	0.28	0.28	0.27
Prop. of adult males (m)	0.12	0.15	0.10	0.24	0.15	0.16
Prop. of adult females (f)	0.54	0.56	0.62	0.48	0.57	0.57
Birth rate (b)	0.23	0.33	0.38	0.18	0.29	0.28
Death rate (d)	0.15	0.31	0.32	0.11	0.19	0.19
Offtake rate (θ)	0.08	0.02	0.06	0.07	0.10	0.09

* Median number of animals kept per household.

Step C: Production in the presence of brucellosis

Data on a range of production variables for each livestock species and production system were obtained from LSMS and RHoMIS datasets and used to inform model parameters.

The monetary variables originally recorded in the local currency (Tanzania Shillings) were converted to international dollars (int. $) based on purchasing power parity, using the year of data collection and the World Bank conversion rates for Tanzania (available at `https://data.worldbank.org`).

Given the scarce data on milk-related variables and the collection of data on these variables for sheep and goats together in LSMS, the proportion of households that harvested milk, the milk yield, and the lactation length were obtained as common values from both species in LSMS and RHoMIS.

The system-specific production data in the presence of brucellosis were obtained as follows (for each species, except ox and dung usage, which applied only to cattle):

- Wholesale price (w):

  The values of w, the price of livestock, in Tanzania Shillings, at farm gate, were obtained from LSMS and RHoMIS. To calculate these values for each household, the income generated from the sale of live animals during the 12 months preceding the interview was divided by the number of animals sold within the same period.

- Milk harvest (i):

  The values of i, the proportion of households that harvest milk (for consumption or sale), were obtained from LSMS and RHoMIS. To calculate these values, the number of households that reported harvesting milk from livestock at least once in the 12 months preceding the interview was divided by the number of households that kept livestock at the time of the interview.

- Milk yield (y):

  The values of y, the volume of milk, in litres, produced daily per milked animal, were obtained from LSMS and RHoMIS through distinct ways. In LSMS, these values were obtained as reported by each household that harvested milk. In

RHoMIS, each household reported the volume of milk produced in the 'good season' and in the 'bad season', as defined by the respondent, at the herd or individual level. Therefore, the values of y were calculated as the milk production in the 'good season', plus the milk production in the 'bad season', divided by two. These values were further divided by the number of animals milked if the volume reported by the household was at the herd level.

- Lactation length (l):

  The values of l, the number of days on average that animals were milked for, were obtained from LSMS only. This variable was available from the dataset in units of months. To convert it into days, the variable was multiplied by 30.

- Ox usage (o):

  The value of o, the average area ploughed per year, in acres, by each ox owned, was obtained from LSMS only. To calculate this value, the total area of land owned or cultivated by households that reported using animals for ploughing the fields in the 12 months preceding the interview was divided by the total number of bulls or steers kept at the time of the interview (at survey level, i.e., including all households in each production system).

- Dung usage (u):

  The value of u, the average quantity of dung used, in kilograms, per animal per year, was obtained from LSMS only. To calculate this value, the total quantity of dung used for any purpose (e.g., manure, cooking fuel, feed to other animals, and construction) in the 12 months preceding the interview was divided by the total number of animals kept at the time of the interview (at survey-level, i.e., including all households in each production system).

The production parameter values used in each iteration of the production losses model were sampled from distributions fitted to LSMS and/or RHoMIS data by maximum likelihood, using the fitdistrplus package in R [13], with some exceptions:

- the milk harvest (i) was used as a beta distribution with shape parameters ($\alpha = k + 1$, $\beta = n - k + 1$), where k and n are the number of households that reported harvesting milk from livestock at least once in the 12 months preceding the interview and the number of households that kept livestock at the time of the interview, respectively [14];

- the ox and dung usage (o and u, respectively) entered the model as fixed values - some uncertainty around the draught power and dung values was accounted for in the uniform distributions of the prices of oxen hire and dung in step D.

The probability density functions that resulted from fitting distributions and that were sampled from in the model (wholesale price, milk yield, milk price, and lactation length) are shown in Table C.

The production characteristics reported by households in the RHoMIS and LSMS surveys are shown in Table D. For sheep and goats, the milk-related variables (milk harvest - i, milk yield - y, and lactation length - l) combine data for the two species.

Only a small number of differences between production systems were identified. The milk yield of cattle in the pastoral system (median = 1.3 L/day) was significantly lower (p-value < 0.001) than the yield reported by non-pastoral households (median = 3.0 L/day). The milk yield of sheep and goats did not differ between production systems, but pastoral households were significantly more likely (p-value < 0.001) to harvest milk

Table C: Probability density functions used to parametrise the production losses model. Int. \$: international dollars.

Species	Parameter	Production system Pastoral	Non-pastoral
Cattle	Wholesale price (w, int. \$)	lnorm(6.21, 0.50)	gamma(3.15, 0.01)
	Milk yield (y, litres per day)	lnorm(0.34, 0.79)	lnorm(0.94, 0.98)
	Milk price (p, int. \$/L)	gamma(2.04, 2.39)	gamma(2.04, 2.39)
	Lactation length (l, days)	nbinom(3.95, 208.65)	nbinom(3.48, 198.29)
Sheep	Wholesale price (w, int. \$)	gamma(5.24, 0.09)	gamma(3.84, 0.08)
	Milk yield (y, litres per day)	gamma(0.98, 1.11)	gamma(1.85, 1.43)
	Lactation length (l, days)	nbinom(2.77, 161.74)	nbinom(2.50, 106.69)
Goats	Wholesale price (w, int. \$)	gamma(5.35, 0.10)	gamma(4.64, 0.08)
	Milk yield (y, litres per day)	gamma(0.98, 1.11)	gamma(1.85, 1.43)
	Lactation length (l, days)	nbinom(2.77, 161.74)	nbinom(2.50, 106.69)

'lnorm' refers to the log-normal distribution, with parameters μ and σ, which are the mean and standard deviation, respectively; 'gamma' refers to the gamma distribution, with parameters α (or shape) and β (or scale or 1/rate), respectively; 'nbinom' refers to the negative binomial distribution, with parameters 'size' and μ, which are the dispersion parameter and the mean, respectively.

(29 out of 132, 22%) from these species than non-pastoral households (23 out of 591, 3.9%).

Regarding the ox and cattle dung usage variables, the values reported by non-pastoral households were approximately twice as high as those reported by pastoral households.

Table D: Production characteristics in the presence of brucellosis, by production system (pastoral and non-pastoral) and species (cattle, sheep, and goats), as calculated from the LSMS and RHoMIS datasets.

	Pastoral						Non-pastoral					
	Cattle		Sheep		Goats		Cattle		Sheep		Goats	
	Value	n	Value	n	Value	n	Value	n	Value	n	Value	n
Wholesale price (w; int. $)	467 (207-1,389)	145	57 (19-103)	20	57 (16-99)	41	464 (117-1,170)	351	42 (8-109)	65	54 (11-110)	181
Milk harvest (i; %)	82.3	192	22.0	132	22.0	132	76.6	725	3.9	591	3.9	591
Milk yield (y; L/day)	1.3 (0.3-8.0)	157	1.0 (0.0-2.6)	30	1.0 (0.0-2.6)	30	3.0 (0.3-15.0)	522	1.0 (0.1-3.0)	20	1.0 (0.1-3.0)	20
Lactation length (l; days)	180 (60-360)	67	120 (46-360)	23	120 (46-360)	23	180 (30-360)	352	105 (30-197)	18	105 (30-197)	18
Ox usage (o; acres)	1.3	78	NA		NA		2.6	490	NA		NA	
Dung usage (u; kg)	59.7	78	NA		NA		102.7	490	NA		NA	

For the w, y, and l, the values shown are the median and, inside parenthesis, the 95% uncertainty interval. 'n' indicates the number of households from which values were obtained. 'NA' indicates a parameter that is not applicable to a species. The data on i, y, and l for sheep and goats combines the two species. Int $: international dollars. L/day: litres per day.

Step D: Global parameters

Global parameters covering information on direct brucellosis impacts and some production characteristics were gathered from the literature, except the milk price, which was gathered from LSMS and RHoMIS. The same set of parameter values was used across production systems. The available data on direct brucellosis impacts and some production characteristics (milk price, litter size, ox hire, and dung price) was not sufficient to partition them by production system.

To gather information on direct brucellosis impacts, a systematic search of the literature was carried out through backward and forward chaining, as described by Hinde and Spackman (2015) [15]. References and citation indices were used to find documents that cited or were cited by two recent studies that estimated losses attributable to livestock brucellosis: Alves et al. (2015) [16] and Singh et al. (2015) [17]. Once the initial pool of references was scanned for brucellosis impacts in livestock, the process was repeated to gather additional references and citations until no new relevant documents were found. Additional targeted searches were carried out to identify further references. Minimum and maximum values of the following model parameters reported in the literature were used to set up uniform distributions of brucellosis impacts:

- Probability of abortion in infected females that have become pregnant (δ);
- Probability of infected females being (temporarily) infertile (η; a fixed value was used, rather than a uniform distribution, because a period of two months per year was consistently reported in the literature as the duration of infertility of infected females);
- Probability of infected females becoming sterile (permanently infertile) after an abortion event (γ);
- Proportional increase in perinatal mortality rate in offspring from infected females (ν);
- Proportional increase in mortality rate of infected females that aborted (ψ);
- Proportional reduction in daily milk yield in infected animals (ϕ);
- Proportional reduction in meat production in infected animals (ω);
- Proportional reduction in draught power in infected oxen (ξ).

The following parameter values on some production characteristics were used across production systems:

- Milk price (p):

  The values of p, the price of milk, in Tanzania Shillings per litre, at farm gate, were obtained from LSMS and RHoMIS, differently for cattle and sheep and goats.

  In cattle, distributions were fitted to data on milk price by maximum likelihood, as described for parameters in step B. The data in LSMS and RHoMIS were processed through distinct ways, as follows:

  – In LSMS, the daily income from sales of milk in the 12 months preceding the interview included income from sales of both liquid milk and processed dairy products. Therefore, only households that reported not converting milk into processed dairy products were used in the calculation of p. The values of p were obtained by dividing the daily income from sales of milk in the 12 months preceding the interview by the volume of milk, in litres, sold per day in the same period.

- In RHoMIS, the volume of milk sold is recorded in semi-quantitative terms (e.g., ‘all’, ‘most’, ‘half’, ‘little’, ‘under half, ‘none’) and the income from milk sales is recorded in several units of time (e.g., daily, weekly, monthly, yearly). Additionally, no information on lactation length is provided in RHoMIS. Therefore, only responses on income from milk sales that were recorded in daily time units and related to ‘large’ volumes (i.e., ‘all’, ‘most’, or ‘half’) were used to calculate p. It was assumed that ‘all’, ‘most’, and ‘half’ referred to 100%, 75%, and 50% of the average volume of milk produced daily, respectively. The values of p were obtained by dividing the daily income from milk sales by the average volume of milk sold per day.

  Given the scarce data on milk sales in the study area, particularly on sales of sheep and goat milk, and the fact that milk-related variables were collected for sheep and goats together in LSMS, the approach for gathering data on milk price for sheep and goats combined was different from that of cattle. A PERT distribution was parametrised with the sheep and goat milk prices available in the unfiltered (i.e., unrestricted to the study area) RHoMIS1 and RHoMIS2 datasets [18].

- Litter size (z):

  The values of z, the average number of offspring per term pregnancy, were obtained from the literature using the same method as described for the direct brucellosis impacts. Searches were also carried out to identify references specifically from Tanzania. Given that twining is a relatively rare event in cattle in Tanzania, a fixed value of 1 was used in this species model. In the goats and sheep models, uniform distributions were set up with values obtained from the literature.

- Ox hire (h):

  The values of h, the cost of hiring oxen for ploughing one acre of land, in Tanzania Shillings, were obtained from the literature through ‘Google’ searches, using the search string ‘ox*’, ‘hir*’, and ‘Tanzania’. Uniform distributions were set up with these values obtained from the literature.

- Dung price (g):

  The values of g, the price of dung, in Tanzania Shillings per kilogram, were obtained from the literature through ‘Google’ searches, using the search string ‘price’, ‘dung’, ‘manure’, and ‘Tanzania’. Uniform distributions were set up with these values obtained from the literature.

The uniform distributions used for global parameters are shown in Table E. In addition, the following median prices (95% uncertainty interval) of milk (p) were used:

- cattle milk = 0.81 (0.13-2.65) int. $/L; and,
- sheep and goats milk = 0.55 (0.36-0.74) int. $/L.

Step E: Demography in the absence of brucellosis

The data described in steps A, B, and C were used to estimate the full-term pregnancy rate (α) and the age- and sex-specific death rates (μ) that would be expected in the absence of brucellosis. Note that b (in step B) is the number of animals born divided by the number of animals kept, in the presence of brucellosis, whilst α is the proportion of adult females that are expected to complete a full-term pregnancy each

Table E: Range of values (minimum and maximum) identified in the literature and used to parametrise uniform distributions of global parameters.

Global parameters	Minimum	Maximum	References
Abortion probability (δ)	0.100	0.500	[19, 20]
Sterility probability (γ)	0.100	0.300	[17, 19, 21]
Proportional increase in perinatal mortality (ν)	0.050	0.200	[16, 17, 19–22]
Proportional increase in mortality due to abortion (ψ)	0.005	0.015	[16, 17, 19]
Proportional reduction in milk yield (ϕ)	0.100	0.250	[16,17,19,20,23,24]
Proportional reduction in meat production (ω)	0.000	0.150	[16, 17, 19–21, 24]
Proportional reduction in draught power (ξ)	0.000	0.040	[17]
Litter size (z) in sheep	1.020	1.180	[25, 26]
Litter size (z) in goats	1.100	1.640	[27, 28]
Ox hire (h; int. $)	35.40	56.60	[29]
Dung price (g; int. $)	0.010	0.040	[30]

year in the absence of brucellosis, assuming one breeding season per year. It was also assumed that each full-term pregnancy leads to the birth of z (litter size; step D) live offspring. The α and μ parameters were estimated as follows:

$$\begin{aligned}\alpha &= \frac{bz^{-1}N}{((1-\pi)+\pi(1-\delta-\eta-\gamma^*))F}\\ \mu_J &= \frac{dJ}{J+\nu\pi\alpha(1-\delta-\eta-\gamma^*)zF}\\ \mu_M &= d\\ \mu_F &= \frac{d}{1+\psi\pi\alpha\delta(1-\eta-\gamma^*)}\end{aligned} \tag{2}$$

where N is the sum of the number of juveniles (J), adult males (M), and adult females (F) in the modelled herd; the subscripts J, F, and M in μ indicate sex- and age-specific mortality rates; and, γ^* is the proportion, in equilibrium, of infected females that are sterile due to a previous abortion event. γ^* was calculated assuming that the disease is in endemic equilibrium and the number of sterile females ($S = \gamma^*\pi F$) is constant over time, so that $\frac{dS}{dt}$ in Eq. 3 is equal to zero:

$$\frac{dS}{dt} = \gamma\delta\alpha((1-\eta)\pi F - S) - (d+\theta)S \tag{3}$$

It was assumed that the death and offtake rates of sterile females were not different from those of the general population.

Given the equation for α (Eq. 2) and the assumption of endemic equilibrium, $\frac{dS}{dt} = 0$, and that $\theta = b - d$, Eq. 3 can be simplified as follows:

$$0 = \frac{\gamma\pi\delta(1-\eta-\gamma^*)bz^{-1}N}{(1-\pi)+\pi(1-\delta-\eta-\gamma^*)} - b\pi\gamma^*F \tag{4}$$

Eq. 4 can then be solved for γ^* as follows, knowing that $F = fN$ and $0 \leq \gamma^* < 1$:

$$\gamma^* = \frac{(1-\pi(\delta+\eta))f + \gamma\delta z^{-1} - \sqrt{((1-\pi(\delta+\eta))f + \gamma\delta z^{-1})^2 - 4\pi\gamma\delta(1-\eta)z^{-1}f}}{2\pi f} \tag{5}$$

Step F: Production in the absence of brucellosis

The value of production per animal (offtake, milk, and, in the case of cattle, draught power) that would be expected in the absence of brucellosis was estimated. It was assumed that infected animals were less valuable due to their lower weight, lower milk yield, and lower draught power. These three direct impacts of brucellosis were accounted for in the following model parameters:

- Value of offtake per animal in the absence of brucellosis (Ω):

  Ω is the income, or the equivalent monetary metric of utility, gained by a household, in the absence of brucellosis, from each animal that is sold, slaughtered or given away as a gift. It was assumed that brucellosis causes a proportional reduction in meat production (and live weight) in infected animals (ω; step D). However, data on the live weight of animals at the moment of sale were not available in the survey datasets, so a linear relationship between live weight and wholesale price (w) was assumed.

- Value of milk per lactation in the absence of brucellosis (Φ):

  Φ is the income, or the equivalent monetary metric of utility, gained by a household, in the absence of brucellosis, from the milk produced by each adult female in one lactation period. It was assumed that each pregnancy was followed by a lactation period, i.e., the reduction in milk production by infected females that conceived each year was only affected by the parameter ϕ (step D).

- Value of draught power per ox in the absence of brucellosis (Ξ):

  Ξ is the income, or the equivalent monetary metric of utility, gained by a household, in the absence of brucellosis, from using an ox for ploughing an average area of land (o; step D) each year.

The parameters Ω, Φ, and Ξ were calculated as follows:

$$\begin{aligned}\Omega =& \frac{w}{(1-\pi)+\pi\,(1-\omega)}\\ \Phi =& ylp\frac{(1-\pi)+\pi\,(1-\eta-\gamma^*)}{(1-\pi)+\pi\,(1-\eta-\gamma^*)\,(1-\phi)}\\ \Xi =& \frac{ho}{(1-\pi)+\pi\,(1-\xi)}\end{aligned} \tag{6}$$

Step G: Production simulations

To model the effects of brucellosis on livestock reproduction and mortality (lower birth rate, higher perinatal mortality, and higher female mortality), a set of differential equations was used (Eqs. 7). These differential equations enabled estimating the number of animals in each age and sex group and the number of events (births, deaths, abortions, etc.) at any point in time for the duration of the simulation (10 years). Animals could (i) leave the system through death (at a rate μ), or offtake (at a rate θ), and (ii) enter the system through birth (at a rate α).

$$
\begin{aligned}
\frac{dJ}{dt} =& \left(\left(1-\pi\right) + \pi\left(1-\delta-\eta-\gamma^{*}\right)\right)\alpha z F - \\
& \mu_J\left(J + \nu\pi\left(1-\delta-\eta-\gamma^{*}\right)\alpha z F\right) - \left(\theta+\sigma_M+\sigma_F\right)J \\
\frac{dM}{dt} =& \sigma_M J - \left(\mu_M+\theta\right)M \\
\frac{dF}{dt} =& \sigma_F J - \left(\mu_F\left(1+\psi\pi\alpha\delta\left(1-\eta-\gamma^{*}\right)\right)+\theta\right)F
\end{aligned}
\tag{7}
$$

where π is the prevalence of brucellosis (constant for all age and sex groups), and, σ_F and σ_M are the transition rates from juveniles to adult females and adult males, respectively. Given that sex-specific mortality and offtake rates for juveniles were not available in SEEDZ, the transition rates σ_F and σ_M were updated numerically to ensure a stable sex structure in the livestock population over time.

The household production values, i.e., income or utility (e.g., in the form of livestock given away as gifts) from offtake, milk, and, in cattle, from draught power and dung, were estimated for the period of ten years, before and after the elimination of brucellosis from the system. The dung value only changed upon brucellosis elimination in the model scenario of 'unrestricted increase in herd size'. These estimates of household production were obtained from the following set of equations:

$$
\begin{aligned}
\text{Milk value} =& \left(\left(1-\pi\right)+\pi\left(1-\eta-\gamma^{*}\right)\left(1-\phi\right)\right)\Phi\alpha i F \\
\text{Offtake value} =& \left(\left(1-\pi\right)+\pi\left(1-\omega\right)\right)\Omega\theta N \\
\text{Draught power value} =& \left(\left(1-\pi\right)+\pi\left(1-\xi\right)\right)\Xi M \\
\text{Dung value} =& ugN
\end{aligned}
\tag{8}
$$

All monetary values were discounted over time (t, in years) at a rate (r) of 3%, as per Eq. 9:

$$
\text{Discounted value} = \frac{\text{Non-discounted value}}{\left(1+r\right)^{t}} \tag{9}
$$

The losses due to brucellosis per household, per year, were estimated as the difference in production values between the periods before and after disease elimination ($\sum$, red shaded area in box G of Fig 1 in the main text, indicating cumulative livestock production over time), divided by the duration of the simulation ($t = 10$ years).

The losses per infected animal, per year, were estimated as the losses per household, divided by the number of infected animals within the household (πN) at the start of the simulation.

The losses per region, per year, were estimated as the losses per animal kept (losses per household divided by the number of animals kept, N), multiplied by the livestock population in each region, for each species and production system. The distribution of the number of livestock by species, production system, and region is shown in Table F.

Table F: Estimated number of livestock by region, production system (pastoral and non-pastoral), and species (cattle, sheep, and goats), in northern and central Tanzania.

	Pastoral			Non-pastoral		
Region	Cattle	Sheep	Goats	Cattle	Sheep	Goats
Arusha	808,849	503,569	2,008,281	364,595	155,649	571,736
Dodoma	636,560	140,920	565,250	1,175,867	178,498	659,458
Kilimanjaro	38,654	12,632	49,755	755,370	169,266	614,088
Manyara	1,321,002	304,687	1,252,187	880,668	139,286	527,236
Mara	98,076	27,091	69,025	2,059,591	390,107	915,490
Simiyu	112,683	57,412	124,713	1,659,734	579,857	1,160,158
Singida	109,872	26,362	108,062	1,762,530	289,977	1,094,837
Tanga	286,377	76,996	284,424	918,791	169,390	576,333

The total number of animals in each region for each species was extracted from the report of the Annual Agricultural Sample Survey 2016-2017 (Government of The United Republic of Tanzania, 2017) [31]. The values in this table are based on the following assumptions: i) the proportion of households that belong to pastoral and non-pastoral production systems in each region are the same as those in the LSMS household classifications, and, ii) the median number of animals of each species kept by each household and the distribution of brucellosis prevalence by production system are the same as in the SEEDZ survey.

References

1. Bodenham RF, Mazeri S, Cleaveland S, Crump JA, Fasina FO, de Glanville WA, et al. Latent class evaluation of the performance of serological tests for exposure to *Brucella* spp. in cattle, sheep, and goats in Tanzania. PLoS Neglected Tropical Diseases. 2021;15(8):e0009630. doi:10.1371/journal.pntd.0009630.

2. Bronsvoort BM, Koterwas B, Land F, Handel IG, Tucker J, Morgan KL, et al. Comparison of a flow assay for brucellosis antibodies with the reference cELISA test in West African *Bos indicus*. PloS One. 2009;4(4). doi:10.1371/journal.pone.0005221.

3. Toft N, Jørgensen E, Højsgaard S. Diagnosing diagnostic tests: evaluating the assumptions underlying the estimation of sensitivity and specificity in the absence of a gold standard. Preventive Veterinary Medicine. 2005;68(1):19–33. doi:10.1016/j.prevetmed.2005.01.006.

4. Mazeri S, Sargison N, Kelly RF, Bronsvoort BM, Handel I. Evaluation of the performance of five diagnostic tests for *Fasciola hepatica* infection in naturally infected cattle using a Bayesian no gold standard approach. PloS One. 2016;11(8):e0161621. doi:10.1371/journal.pone.0161621.

5. Branscum AJ, Gardner IA, Johnson WO. Estimation of diagnostic-test sensitivity and specificity through Bayesian modeling. Preventive Veterinary Medicine. 2005;68(2-4):145–163. doi:10.1016/j.prevetmed.2004.12.005.

6. Hui SL, Walter SD. Estimating the error rates of diagnostic tests. Biometrics. 1980;36(1):167–171.

7. Vacek PM. The effect of conditional dependence on the evaluation of diagnostic tests. Biometrics. 1985;41(4):959–968. doi:10.2307/2530967.

8. Dendukuri N, Joseph L. Bayesian approaches to modeling the conditional dependence between multiple diagnostic tests. Biometrics. 2001;57(1):158–167. doi:10.1111/j.0006-341x.2001.00158.x.

9. R Core Team. R: A language and environment for statistical computing. R Foundation for Statistical Computing, Vienna, Austria. Version 3.6.2. 2019.

10. Plummer M. JAGS: A program for analysis of Bayesian graphical models using Gibbs sampling. International Workshop on Distributed Statistical Computing. 2003; p. 1–10.

11. Plummer M, Stukalov A, Denwood M. rjags: Bayesian graphical models using MCMC. Version 4-9. 2019.

12. Plummer M, Best N, Cowles K, Vines K, Sarkar D, Bates D. coda: Output Analysis and Diagnostics for MCMC. Version 0.19-3. 2019.

13. Delignette-Muller ML, Dutang C. fitdistrplus: An R package for fitting distributions. Journal of Statistical Software. 2015;64(4):1–34.

14. Chan BKC. Biostatistics for epidemiology and public health using R. New York, New York: Springer Publishing Company; 2016.

15. Hinde S, Spackman E. Bidirectional citation searching to completion: an exploration of literature searching methods. Pharmacoeconomics. 2015;33(1):5–11. doi:10.1007/s40273-014-0205-3.

16. Alves AJS, Rocha F, Amaku M, Ferreira F, Telles EO, Filho JHHG, et al. Economic analysis of vaccination to control bovine brucellosis in the States of São Paulo and Mato Grosso, Brazil. Preventive Veterinary Medicine. 2015;118(4):351–358. doi:10.1016/j.prevetmed.2014.12.010.

17. Singh BB, Dhand NK, Gill JPS. Economic losses occurring due to brucellosis in Indian livestock populations. Preventive Veterinary Medicine. 2015;119(3):211–215. doi:10.1016/j.prevetmed.2015.03.013.

18. Bergevoet RH, van Schaik G, Veling J, Backus GB, Franken P. Economic and epidemiological evaluation of *Salmonella* control in Dutch dairy herds. Preventive Veterinary Medicine. 2009;89(1-2):1–7.

19. Bernues A, Manrique E, Maza MT. Economic evaluation of bovine brucellosis and tuberculosis eradication programmes in a mountain area of Spain. Preventive Veterinary Medicine. 1997;30(2):137–149. doi:10.1016/s0167-5877(96)01103-8.

20. Astaiza-Martínez JM, Benavides-Melo JC, Díaz-Rojas JA. Estudio de costo-efectividad del programa de vacunación contra *Brucella abortus* en bovinos en el departamento de Nariño. Revista Colombiana de Ciencias Químico - Farmacéuticas. 2012;41(2):167–186.

21. Capdevilla MJM. La brucelosis en la provincia de Huesca (estado actual y repercusion economica). vol. 31 of Colección de Estudios Altoaragoneses. Zaragoza: Instituto de estudios Altoaragoneses; 1989.

22. Hugh-Jones ME, Ellis PR, Felton MR. An assessment of the eradication of bovine brucellosis in England and Wales. Department of Agriculture and Horticulture, University of Reading; 1975.

23. Carpenter TE. The application of benefit-cost analysis to compare alternative approaches to the brucellosis problem in California: new techniques in veterinary epidemiology and economics. 1976.

24. Shepherd A, Simpson B, Davidson R. An economic evaluation of the New Zealand bovine brucellosis eradication scheme. In: Proceedings of the Second International Symposium on Veterinary Epidemiology and Economics; 1980. p. 443–447.

25. Tungu GB, Kifaro GC, Gimbi AA, Mashingo M, Nguluma AS. Effect of genetic and non-genetic factors on growth and reproduction performance of Black Head Persian and Red Masai Sheep in Tanzania. International Journal of Veterinary Sciences and Animal Husbandry. 2017;2(5):4–10.

26. Kosgey IS, van Arendonk JAM, Baker RL. Economic values for traits of meat sheep in medium to high production potential areas of the tropics. Small Ruminant Research. 2003;50(1):187–202. doi:10.1016/S0921-4488(03)00102-0.

27. Safari J, Mtenga LA, Eik LO, Sundstøl F, Johnsen FH. Analysis of three goat production systems and their contribution to food security in semiarid areas of Morogoro, Tanzania. Livestock Research for Rural Development. 2008;20(5):e74.

28. Dadi H, Duguma G, Shelima B, Fayera T, Tadesse M, Woldu T, et al. Non-genetic factors influencing post-weaning growth and reproductive performances of Arsi-Bale goats. Livestock Research for Rural Development. 2008;20(7):1–10.

29. Mutabazi KD. Identifying, defining and typologizing food value chains and upgrading strategies: A qualitative scoping study. Innovating pro-poor strategies to safeguard food security using technology and knowledge transfer. Deliverable 3.1.1. Sokoine University of Agriculture, Tanzania. 2014.

30. Kangondo A. Economics of manure disposal and utilization in Morogoro Municipality, Tanzania. Sokoine University of Agriculture. Master Thesis; 2015.

31. Government of The United Republic of Tanzania. 2016/17 Annual agriculture sample survey. Crop and livestock report. Ministry of Agriculture; Ministry of Livestock and Fisheries; Ministry of Industry, Trade and Investment; President's Office, Regional Administration and Local Governments; Ministry of Agriculture, Natural Resources, Livestock and Fisheries, Zanzibar; National Bureau of Statistics; and the Office of the Chief Government Statistician, Zanzibar. 2017. Available from: https://www.nbs.go.tz/nbs/takwimu/Agriculture/2016-17_AASS%20Report%20_Final.pdf.
