# Supplementary material for: Livestock production losses attributable to brucellosis in northern and central Tanzania: Application of an epidemiological-economic modelling framework
Source: PLoS Negl Trop Dis. 2025 Feb 14;19(2):e0012814. doi: 10.1371/journal.pntd.0012814 (PMC11828364; doi:10.1371/journal.pntd.0012814)
Supplement: S4 File — (PDF) [file pntd.0012814.s004.pdf]

Livestock production losses attributable to brucellosis in northern and central Tanzania: application of an epidemiological-economic modelling framework

Ângelo J. F. Mendes[1*], Daniel T. Haydon[1], William A. de Glanville[1], Rebecca F. Bodenham[1], AbdulHamid S. Lukambagire[2], Paul C. D. Johnson[1], Gabriel M. Shirima[3], Sarah Cleaveland[1], Emma McIntosh[4], Nick Hanley[1], Jo E. B. Halliday[1]

1 School of Biodiversity, One Health and Veterinary Medicine, College of Medical, Veterinary and Life Sciences, University of Glasgow, Glasgow, United Kingdom
2 Kilimanjaro Clinical Research Institute, Kilimanjaro Christian Medical University College, Moshi, Tanzania
3 School of Life Sciences and Bioengineering, The Nelson Mandela African Institution of Science and Technology, Arusha, Tanzania
4 School of Health and Wellbeing, College of Medical, Veterinary and Life Sciences, University of Glasgow, Glasgow, United Kingdom

* a.mendes.1@research.gla.ac.uk

Supporting information

S4 File. Determinants of production losses attributable to brucellosis

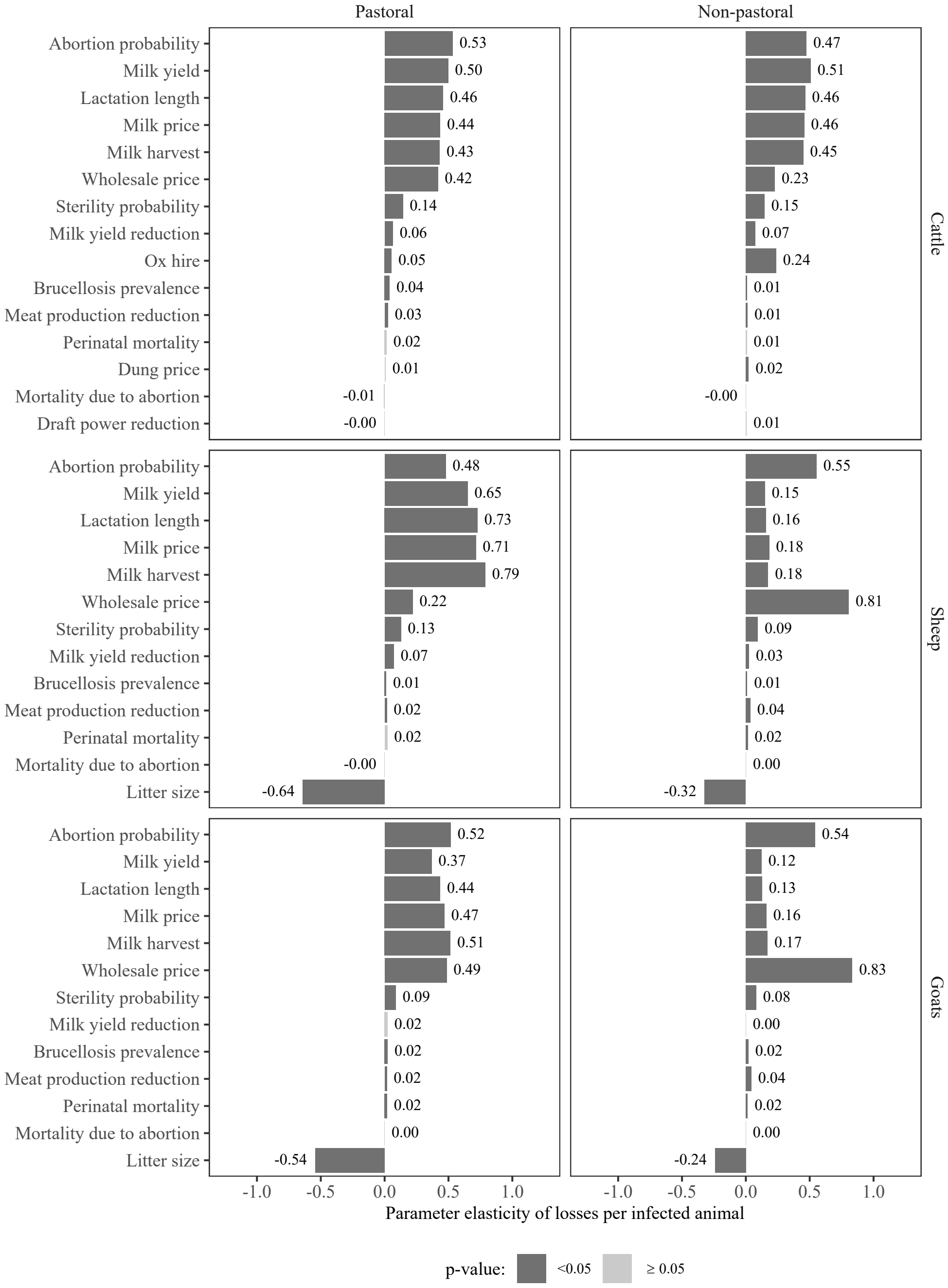

Figure A: Tornado plot showing the parameter elasticity of production losses per infected animal (percentage change in production losses per infected animal per year when parameter values change by 1%), by production system (pastoral and non-pastoral) and species (cattle, sheep, and goats) (50,000 model iterations, scenario of 'unrestricted increase in herd size'). The colours of the bars indicate the significance of the parameter elasticity estimate (dark and light grey indicate p-value < 0.05 and p-value ≥ 0.05, respectively).

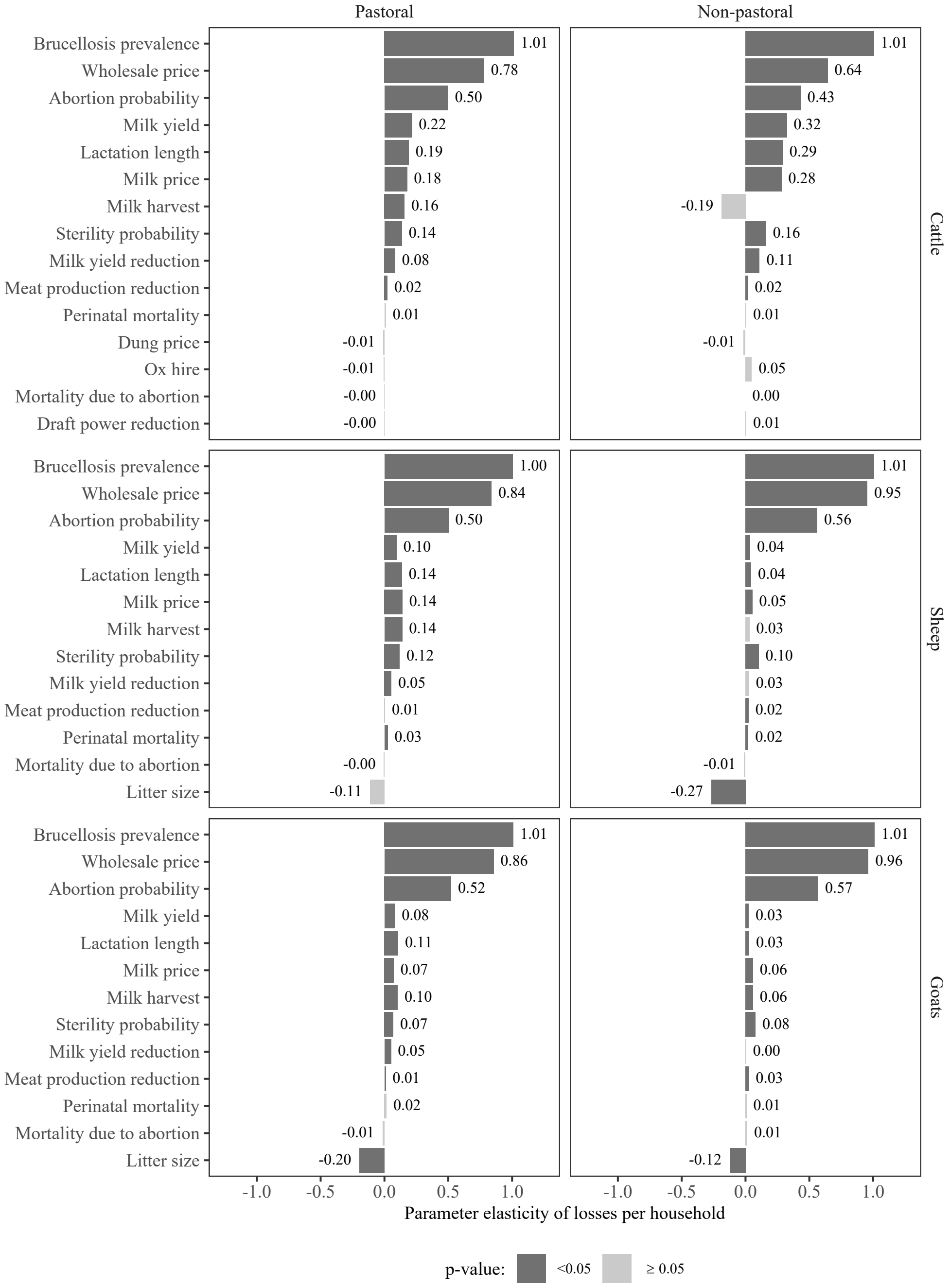

Figure B: Tornado plot showing the parameter elasticity of production losses per household (percentage change in production losses per household per year when parameter values change by 1%), by production system (pastoral and non-pastoral) and species (cattle, sheep, and goats) (50,000 model iterations, scenario of 'no increase in herd size'). The colours of the bars indicate the significance of the parameter elasticity estimate (dark and light grey indicate p-value < 0.05 and p-value ≥ 0.05, respectively).

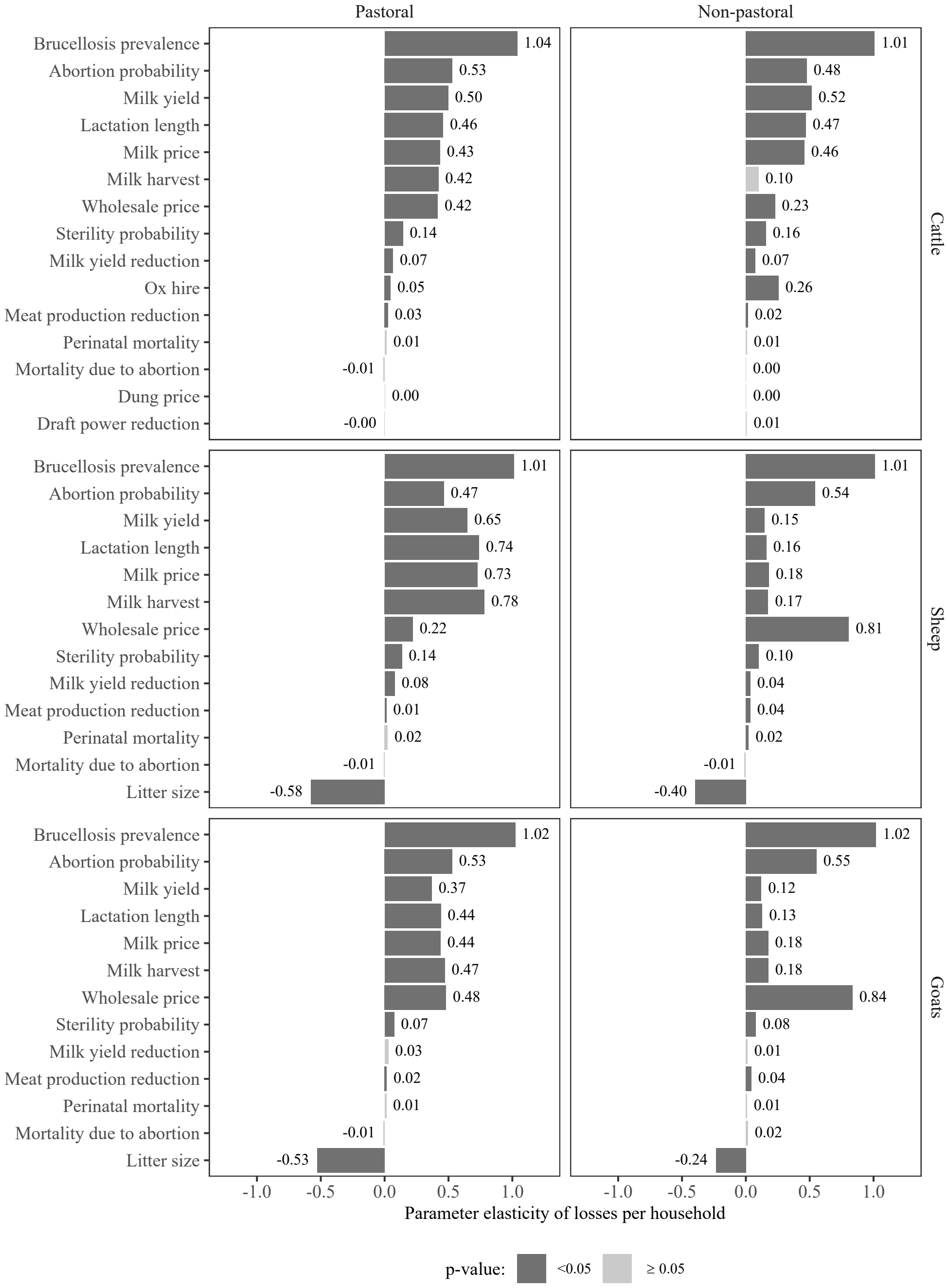

Figure C: Tornado plot showing the parameter elasticity of production losses per household (percentage change in production losses per household per year when parameter values change by 1%), by production system (pastoral and non-pastoral) and species (cattle, sheep, and goats) (50,000 model iterations, scenario of 'unrestricted increase in herd size'). The colours of the bars indicate the significance of the parameter elasticity estimate (dark and light grey indicate p-value < 0.05 and p-value ≥ 0.05, respectively).
